# Carry-over effects between spring and autumn phenology differ among the world’s biomes

**DOI:** 10.1093/nsr/nwag082

**Published:** 2026-02-06

**Authors:** Zhaofei Wu, Yongshuo H Fu, Thomas W Crowther, Susanne S Renner, Yann Vitasse, Lidong Mo, Yibiao Zou, Leila Mirzagholi, Mingwei Li, Dominic Rebindaine, Yufeng Gong, Zhendong Guo, Nan Wang, Constantin M Zohner

**Affiliations:** College of Water Sciences, Beijing Normal University, Beijing 100875, China; Ecosystem Ecology, Swiss Federal Institute for Forest, Snow and Landscape Research (WSL), Birmensdorf 8903, Switzerland; College of Water Sciences, Beijing Normal University, Beijing 100875, China; Department of Biology, University of Antwerp, Antwerpen 2000, Belgium; BRANCH Institute, Zug 6300, Switzerland; Environmental Science and Engineering, King Abdullah University of Science and Technology (KAUST), Thuwal 23955, Saudi Arabia; Department of Biology, Washington University in St. Louis, Saint Louis, MO 63130, USA; Ecosystem Ecology, Swiss Federal Institute for Forest, Snow and Landscape Research (WSL), Birmensdorf 8903, Switzerland; Oeschger Centre for Climate Change Research, University of Bern, Bern 3012, Switzerland; College of Life Science, Nankai University, Tianjin 300071, China; Ecosystem Ecology, Swiss Federal Institute for Forest, Snow and Landscape Research (WSL), Birmensdorf 8903, Switzerland; Institute of Integrative Biology, ETH Zurich, Zurich 8092, Switzerland; Department of Civil and Environmental Engineering, Massachusetts Institute of Technology, Cambridge, MA 02139, USA; College of Water Sciences, Beijing Normal University, Beijing 100875, China; Ecosystem Ecology, Swiss Federal Institute for Forest, Snow and Landscape Research (WSL), Birmensdorf 8903, Switzerland; Institute of Integrative Biology, ETH Zurich, Zurich 8092, Switzerland; College of Water Sciences, Beijing Normal University, Beijing 100875, China; College of Water Sciences, Beijing Normal University, Beijing 100875, China; College of Water Sciences, Beijing Normal University, Beijing 100875, China; BRANCH Institute, Zug 6300, Switzerland; Institute of Integrative Biology, ETH Zurich, Zurich 8092, Switzerland

**Keywords:** climate change, phenological carry-over effects, leaf-out, leaf senescence, physiological constraints

## Abstract

Climate warming alters the start (SOS) and end (EOS) of growing seasons, impacting biotic interactions and biogeochemical cycles, yet the carry-over links between SOS and EOS remain poorly understood, limiting future projections. Using MODIS satellite-derived phenology data for seasonal vegetation and European ground observations for deciduous tree species, we show that an earlier SOS typically advances the EOS [on average by 0.19 ± 0.001 days per day (MODIS) and 0.10 ± 0.002 days per day (ground)], while EOS exerts a weaker influence on subsequent SOS. The SOS-to-EOS effect often outweighed abiotic factors, with SOS emerging as the best predictor of EOS in 34% of pixels (β = 0.27). When predicting SOS, EOS was the primary predictor in only 7.9% of pixels, while preseason temperature dominated in 58% (β = −0.33). More importantly, we identified a dampening interaction, where an increase in one carry-over effect reduced the other. Thus, the SOS-to-EOS effect was twice as strong as the EOS-to-SOS effect in temperate deciduous forests, while the EOS-to-SOS effect was up to three times stronger in boreal taiga and tundra. The observed carry-over effects likely reflect developmental (cell and tissue growth) and stress-related constraints (SOS-to-EOS effect), as well as chilling requirements (EOS-to-SOS effect at high latitudes). These findings highlight how physiological feedback affects phenological responses to climate change, emphasizing the need to integrate plant-internal carry-over effects into future ecosystem models.

## Teaser

This study first identified a widespread phenomenon impacting biotic interactions and biogeochemical cycles. It is found that an earlier start of the growing season (SOS) advanced the end of the growing season (EOS), whereas the effect of EOS on the subsequent year’s SOS was mainly pronounced in high-latitude regions.

## INTRODUCTION

Shifts in plant phenological cycles profoundly influence biotic interactions [1,2], ecosystem dynamics [3–5] and global biogeochemical cycles [6]. Over the past four decades, global warming has advanced the start of the growing season (SOS) in extra-tropical vegetation by an average of 6 to 30 days [4,7,8] and has slightly delayed the end of the growing season (EOS) by 0.1 to 6 days [4,9]. These phenological changes have lengthened growing seasons [10–12], increasing net ecosystem carbon uptake [6,11]. However, the SOS and EOS are not solely driven by environmental factors; they are also influenced by plant-internal mechanisms [13], with potential carry-over effects between the two phenophases [14,15]. Local experiments and observations underscore this interconnection (Table S1), demonstrating that the SOS has profound impacts on the following EOS [15–17], and that the EOS can affect the SOS in the next spring [14].

Phenological carry-over effects are expected to vary regionally because they reflect both species traits and region-specific environmental controls [18–20]. In temperate regions, an earlier SOS often leads to an earlier EOS [15,16,21,22], which has been explained by developmental and nutrient constraints [23,24], limited leaf lifespan [25], pathogen load increases [26], oxidative stress [27,28] and increased drought conditions [29]. In dry and semi-tropical regions, an earlier SOS may similarly advance the EOS, but primarily through faster depletion of available water and earlier soil moisture limitation [30–32]. Supporting this broad climatic modulation, a recent study reported that SOS–EOS coupling is stronger in warmer regions of the Southern Hemisphere than in the Northern Hemisphere, in some cases even exceeding the apparent influence of concurrent climate variability [33].

Carry-over effects also operate from autumn into the following spring—linking EOS to the timing of subsequent SOS—through the dormancy processes that precede budburst. Extra-tropical plants typically pass through two dormancy phases before leaf-out [34]: endodormancy in autumn and winter, which requires chilling to be released [35], followed by ecodormancy in late winter and spring, when heat accumulation triggers leaf-out. If chilling requirements are strong, as is expected at higher latitudes [36,37], a delayed EOS could delay the onset and/or completion of endodormancy, thereby postponing the transition to ecodormancy and ultimately the SOS [14,38]. On the other hand, autumn warming-driven delays in the EOS may also advance the SOS by reducing bud dormancy depth [39] and/or enhancing nutrient resorption [40,41]. Despite these insights, we still lack comprehensive evidence across biomes that quantifies the magnitude and direction of carry-over effects and identifies their key environmental drivers.

**Figure 1.**
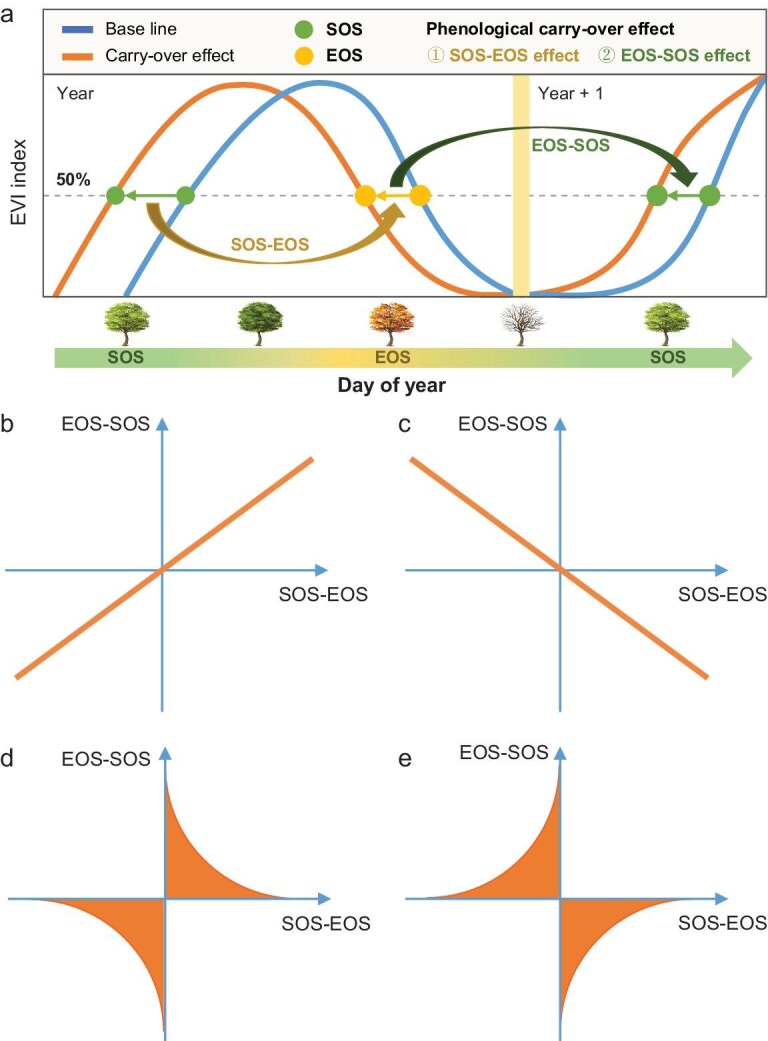
Conceptual models of phenological carry-over effects between the SOS and the EOS across two consecutive years. (a) Conceptual illustration of seasonal carry-over effects, where an earlier SOS can drive an earlier EOS (SOS–EOS effect), and an earlier EOS may advance SOS in the following spring (EOS–SOS effect). Vegetation greenness is shown across two annual cycles [Year and Year + 1] separated by the yellow line, with yellow and green dots indicating the EOS and SOS. (b–e) The *x*-axis represents the magnitude and direction of the SOS–EOS effect, while the *y*-axis represents the EOS–SOS effect for the same pixel. (b) Same direction + reinforcing: both effects act in the same direction, either both positive or both negative. For instance, an earlier SOS advances the EOS, and an earlier EOS advances the following SOS, with stronger effect sizes in one carry-over effect associated with stronger effects in the other (reinforcing). (c) Opposite direction + reinforcing: the two effects act in opposite directions (e.g. an earlier SOS advances the EOS, but an earlier EOS delays the next-year SOS) yet still reinforce each other’s magnitude. (d) Same direction + dampening: both effects act in the same direction, but an increase in one effect reduces the strength of the other (dampening). For example, an earlier SOS leads to an earlier EOS, but an earlier EOS only slightly advances the subsequent SOS. (e) Opposite direction + dampening: the effects act in opposite directions, and an increase in one reduces the impact of the other (e.g. an earlier SOS leads to an earlier EOS, while an earlier EOS slightly delays the next SOS).

In this study, we used satellite-derived and long-term ground-based phenology datasets and applied hierarchical Bayesian and multilinear regression models to quantify the magnitude and direction of SOS–EOS and EOS–SOS phenological carry-over effects in seasonal vegetation (i.e. excluding evergreen vegetation), including boreal forests and tundra, temperate grasslands and forests, and tropical grasslands and savannas. As depicted in our conceptual model, an earlier SOS may drive an earlier EOS (scenario SOS–EOS effect; Fig. 1a) and an earlier EOS may subsequently influence the SOS in the following spring (scenario EOS–SOS effect; Fig. 1a). A key question was whether SOS–EOS and EOS–SOS carry-over effects can coexist simultaneously within the same location and whether the presence of one might influence the other (Fig. 1b–e). One scenario is that both carry-over effects operate simultaneously and reinforce one another, such that a stronger effect in one carry-over pathway (SOS–EOS or EOS–SOS) is associated with a stronger effect in the other (Fig. 1b and c). Alternatively, the two carry-over effects may counteract each other, such that a stronger effect in one direction dampens the other (Fig. 1d and e). In this scenario, regions with a pronounced EOS–SOS carry-over might exhibit a comparatively weaker SOS–EOS effect, and vice versa.

## RESULTS

### Carry-over effects between leaf-out and leaf senescence

**Figure 2.**
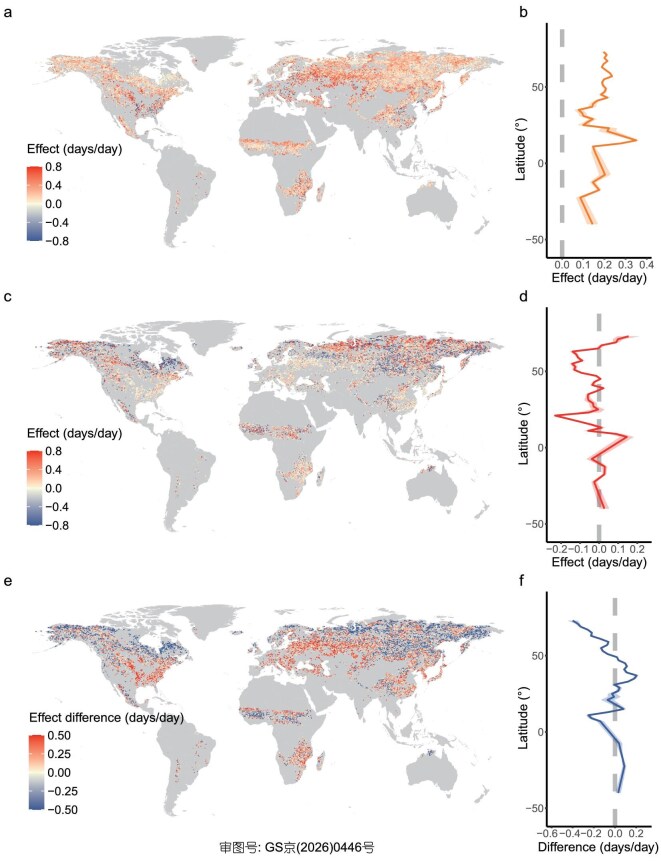
Global carry-over effects between leaf-out and leaf senescence. (a) Map showing the effect of leaf-out onset (SOS_15_) on leaf senescence onset (EOS_10_) (SOS–EOS effect) at 0.25° resolution, derived from multilinear regression models with year and preseason temperature as covariates. (b) Latitudinal variation in the SOS–EOS effect, with solid lines representing mean regression coefficients and shaded areas indicating standard deviations, summarized for each 2° latitude band (bands with fewer than 100 pixels were removed). (c and d) Map and latitudinal variation for the effect of EOS_10_ on SOS_15_ (EOS–SOS effect). (e) Map illustrating the dominant carry-over effect, calculated as the difference between the absolute coefficients of the SOS–EOS and EOS–SOS effects. Red pixels indicate regions where the SOS–EOS effect dominates, while blue pixels indicate a stronger EOS–SOS effect. (f) Latitudinal variation in the relative importance of SOS–EOS versus EOS–SOS effects, with positive values reflecting a stronger SOS–EOS effect and negative values indicating a stronger EOS–SOS effect.

To quantify carry-over effects across global seasonal vegetation, we examined the effects of the SOS on the EOS (SOS–EOS effect) and the EOS on the following SOS (EOS–SOS effect; see Methods). The SOS was estimated as the date when the greenness index first increased by >15% of the seasonal amplitude (SOS_15_; Fig. S1) [42]. The EOS was defined as the date when greenness dropped by 10% (EOS_10_), representing the onset of leaf senescence [42]. We found a positive SOS–EOS effect in 81% of pixels, with 29% of the total pixels showing statistically significant relationships (*P* < 0.05; Fig. 2a, Fig. S2a). On average, each day earlier SOS_15_ advanced EOS_10_ by 0.19 ± 0.001 days (mean ± SE) across all pixels, and by 0.41 ± 0.003 days among significant pixels. This effect was stronger at mid-latitudes, as indicated by the latitudinal pattern in Fig. 2b, particularly in Western Asia, Europe and the southeastern USA (Fig. 2a, Fig. S2a). In contrast, the EOS–SOS effect was weaker and more spatially heterogeneous: 55% of pixels showed negative effects (earlier EOS delays SOS), 45% positive (earlier EOS advances SOS), and only 7% were statistically significant (4% negative and 3% positive; Fig. 2c, Fig. S2c). On average, each day delay in EOS_10_ advanced SOS_15_ by 0.05 ± 0.003 days across all pixels, and by 0.20 ± 0.02 days within the significant subset. The EOS–SOS effect was strongest at high latitudes (Fig. 2c and d, Fig. S2c and S2d). The observed carry-over effects were consistent with previous experimental and regional studies (Table S1).

To compare the relative strengths of the two carry-over effects, we determined the dominant carry-over effect based on the absolute values of their regression coefficients. Globally, the SOS–EOS effect dominated at mid-latitudes, including Europe, Western and Southeast Asia, southeastern North America and South Africa, while the EOS–SOS effect prevailed at high latitudes in the Northern Hemisphere (Fig. 2e and f, Fig. S2e and S2f). These patterns remained consistent when stratifying by vegetation type and when using an alternative EOS metric (EOS_50_; Figs S3–S5), defined as the date when greenness declined by 50% from its seasonal maximum, representing mid-senescence. SOS_15_ had a greater effect on EOS_10_ than on EOS_50_ (Fig. S4).

We also tested the sensitivity of the EOS–SOS effect by replacing SOS_15_ with SOS_50_, defined as the date when greenness first exceeded 50% of the seasonal amplitude. This strengthened the SOS–EOS effect, which dominated across most of the study region, while the magnitude of the EOS–SOS effect declined (Figs S6 and S7). The greater effect of EOS on SOS_15_ than on SOS_50_ likely reflects the closer temporal proximity between the EOS and early spring leaf-out, and the stronger influence of autumn and winter cues on the earlier stages of budburst (Fig. S4e–S4h). Nevertheless, while the later leaf-out stage (SOS_50_) is largely governed by spring temperatures, the preceding EOS still had a detectable influence (Fig. S4g–S4h).

**Figure 3.**
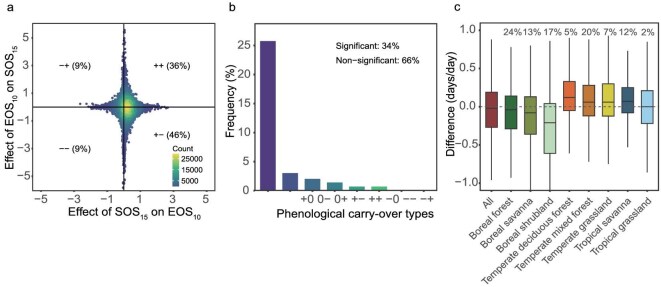
Relationship between the two phenological carry-over effects in seasonally deciduous vegetation. (a) The relationship between the SOS–EOS and EOS–SOS carry-over effects across all pixels. The SOS–EOS effect (SOS_15_ on EOS_10_) and EOS–SOS effect (EOS_10_ on SOS_15_) were estimated using multilinear regression models, with year and preseason temperature as covariates. Percentages in each quadrant represent the proportion of pixels within that category. (b) Frequency distribution of phenological carry-over types. The types are classified by the direction and significance (at *P *< 0.05) of each carry-over effect. For instance, ‘++’ indicates that both effects are positive, while ‘+0’ signifies a positive SOS–EOS effect and a non-significant EOS–SOS effect. Significant carry-over effects were observed in 34% of pixels. (c) The relative strength of each carry-over effect across vegetation types, represented as the difference between SOS–EOS and EOS–SOS effects. The dashed line at 0 indicates equal strength between the two effects, with values below or above 0 indicating a stronger EOS–SOS or SOS–EOS effect, respectively. The percentage values indicate the proportion of pixels for each vegetation type.

To assess how the SOS–EOS and EOS–SOS effects relate to one another, we analyzed pixel-wise correlations between the two. We found that in 55% of pixels, the two effects had opposite directions (Fig. 3a), with the most common pattern being a positive SOS–EOS effect and a negative EOS–SOS effect (46% of pixels). The reverse combination (negative SOS–EOS and positive EOS–SOS) occurred in 9% of pixels. These opposing effect directions were particularly prominent in eastern and northern Asia and North America (Fig. S8). In contrast, 45% of pixels exhibited carry-over effects acting in the same direction, with both effects positive in 36% of pixels and both negative in 9% (Fig. 3a).

We further distinguished nine carry-over types based on the sign and significance of both effects (positive, negative or non-significant). Of the 34% of pixels where at least one effect was significant, the most common carry-over type overall was the ‘+0’ category—indicating a significant positive SOS–EOS effect and a non-significant EOS–SOS effect—found in 26% of all pixels (Fig. 3b). The second most frequent pattern (‘0−’), indicating no significant SOS–EOS effect and a negative EOS–SOS effect occurred in 3% of pixels. Notably, only 2% of pixels showed significant effects for both carry-over types. These results were consistent when substituting EOS_10_ with EOS_50_ and/or SOS_15_ with SOS_50_ (Figs S9–S11). To address uncertainties in enhanced vegetation index (EVI)-derived phenological dates [43,44], we also validated these findings using ground-based phenological observations (1950–2023) from the Pan European Phenology (PEP) network (http://www.pep725.eu/), focusing on four dominant temperate deciduous tree species. These data revealed comparable phenological timing and consistent carry-over patterns between remote-sensing and ground observations (Fig. S12) and independently confirmed the dominance of the ‘+0’ carry-over type (Fig. S13).

Moreover, we observed that an increase in one carry-over effect often coincided with a reduction in the strength of the other (Fig. 3a), a phenomenon we refer to as ‘dampening effect’ (Fig. 1d and e). This pattern was widespread across the studied deciduous vegetation, and remained consistent when using alternative SOS and EOS definitions (Figs S9–S11). In temperate forests and grasslands and tropical woody savannas, the SOS–EOS effect was 1.3–1.8 times stronger than the EOS–SOS effect, corresponding to a difference of 0.07 to 0.15 days per day (Fig. 3c). By contrast, the EOS–SOS effect was most pronounced in boreal forests, savannas and shrublands, where it was 1.4 to 3.3 times (0.09 to 0.42 days per day) stronger than the SOS–EOS effect. These findings support the interpretation that the two carry-over effects tend to balance each other: when one is stronger, the other is typically weaker (dampening effect; Fig. 1d and e).

### Relative contributions of carry-over effects versus environmental cues in predicting leaf senescence and leaf-out

**Figure 4.**
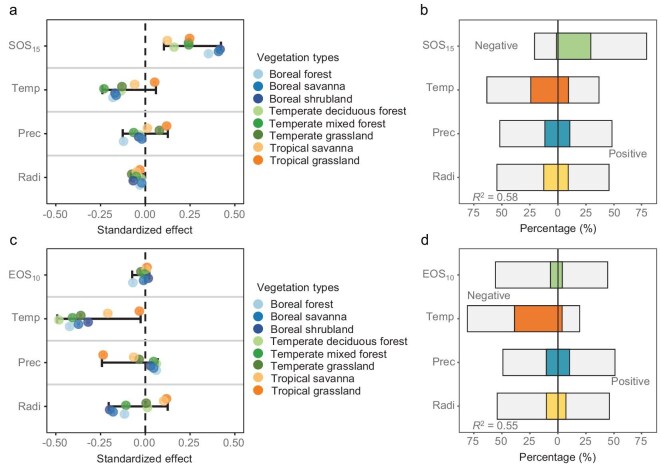
Relative contributions of carry-over effect and environmental factors in driving the onset of leaf senescence (EOS_10_) and leaf-out (SOS_15_). (a and c) Standardized effect sizes of predictor variables—SOS_15_ (a) or EOS_10_ (c) (carry-over effect), preseason temperature (Temp), precipitation (Prec) and radiation (Radi)—on EOS_10_ (a) and SOS_15_ (c), estimated from Bayesian linear mixed-effects models fitted separately for each vegetation type. All predictors were standardized prior to modeling. Pixel was specified as a random effect, and year was modeled as a random slope within pixels to account for temporal variability. Colored points represent posterior means for each vegetation type; black error bars denote the posterior mean and 95% credible interval of biome-level average effects. (b and d) Percentage of pixels where each predictor influenced EOS_10_ (b) or SOS_15_ (d), based on multiple linear regression models. Positive and negative bars represent the percentage of pixels with positive and negative effects, respectively. Colored bars indicate the percentage of significant pixels (*P* < 0.05) for each variable; gray bars show the proportion of non-significant pixels.

To quantify the relative influence of carry-over effects versus environmental factors, including temperature, radiation and precipitation, on phenological timing (EOS or SOS), we identified the optimal preseason windows for each predictor and implemented Bayesian linear mixed-effects and multiple linear regression models [45,46]. The EOS (or SOS) was modeled as the response variable, with the SOS (or EOS from the previous year), preseason temperature, precipitation and radiation as explanatory variables (see Methods). Across the study region, SOS_15_ was the strongest predictor of EOS_10_ in 34% of all pixels (Fig. S14a and S14b), with a posterior mean standardized effect (β = 0.27) more than twice that of preseason temperature (β = −0.13; Fig. 4a). SOS_15_ had a positive effect on EOS_10_ in 79% of pixels, with 29% showing a significant positive effect (Fig. 4b, Fig. S15a). This trend was consistent across vegetation types, with SOS_15_ outperforming climate variables in most cases (Fig. 4a).

The effect of preseason temperature on EOS_10_ displayed more regional variation (Fig. 4a and b, Fig. S15c). Warmer preseason temperatures advanced EOS_10_ in 63% of pixels (24% significantly), particularly in temperate and boreal regions, and delayed EOS_10_ in 37% of pixels (9% significantly), mainly in tropical vegetation (Fig. 4a and b, Fig. S15c). Preseason radiation significantly influenced EOS_10_ in 22% of pixels, advancing it in 13% of those pixels and delaying it in 9% (Fig. 4b, Fig. S15g). Precipitation had a significant effect in 22% of pixels (10% positive, 12% negative), with increased precipitation tending to advance EOS_10_ in high-latitude temperate and boreal forests, but delaying it in temperate and tropical grasslands (Fig. 4a and b, Fig. S15e).

We observed consistent SOS–EOS carry-over effects when using EOS_50_ instead of EOS_10_ to represent the EOS (Fig. S16a and S16b). However, the effects of SOS_15_ on EOS_50_ were weaker than on EOS_10_ (β = 0.20). The effect of preseason temperature on EOS_50_ was predominantly positive, with warming delaying EOS_50_ in 60% of pixels (18% significantly positive, β = 0.09), and this pattern was observed across all vegetation types (Fig. S16a and S16b). The advancing effect of radiation on EOS_50_ was more pronounced in tropical savannas and grasslands (Fig. S16a). Similar patterns were observed when substituting SOS_15_ with SOS_50_ as the predictor (Figs S17 and S18). Ground-based European observations at the species-level further confirmed these trends, identifying SOS_50_ as a stronger predictor of EOS_50_ than radiation and precipitation, but weaker than temperature (Fig. S19a), consistent with the satellite-based results for Europe (Fig. S20a).

In contrast to the EOS, where phenological carry-over effect outweighed the influence of climate, the SOS was more strongly affected by spring climate than by autumn phenological carry-over effect (Fig. 4c, Fig. S14c and S14d). Preseason temperature emerged as the strongest predictor of SOS_15_ in 58% of pixels (β = −0.33; Fig. 4c and Fig. S14c), with higher temperatures advancing SOS_15_ in 81% of pixels (39% significantly; Fig. 4d, Fig. S15d). This advancing effect was most pronounced in temperate and boreal regions (Fig. 4c, Fig. S15d). Precipitation and radiation had smaller and more spatially variable effects. Precipitation significantly influenced SOS_15_ in 21% of pixels, advancing it in 11% and delaying it in 10% (Fig. 4d, Fig. S15f). In subtropical grasslands, where temperature had no significant effect, increased precipitation strongly advanced SOS_15_, indicating water limitation as a key driver (Fig. 4c). Radiation had a significant effect in 17% of pixels, advancing SOS_15_ in 10% and delaying it in 7% (Fig. 4d, Fig. S15h). Despite the critical role of climate variables, the EOS-SOS carry-over effect was the primary predictor in 7.9% of pixels and remained significant in 11% of pixels after accounting for preseason climate (Figs 4d, S14c). However, the direction of this effect was inconsistent (β = −0.01; Fig. 4c and d, Fig. S15b), with delays in EOS_10_ leading to earlier SOS_15_ in 7% of pixels and to later SOS_15_ in 4%.

These patterns were consistent when using EOS_50_ to define the EOS (Fig. S16c and S16d) and were further supported by ground-based, species-level analyses (Fig. S19b). The advancing effect of preseason temperature on the SOS was more pronounced when using SOS_50_ instead of SOS_15_ to represent the SOS (Fig. S17c and S17d, Fig. S18c and S18d). To test whether later stages of leaf senescence have a stronger influence on SOS_15_ and SOS_50_, we replaced EOS_10_/EOS_50_ with EOS_85_, defined as the date when greenness dropped by 85%. However, the EOS–SOS effect remained minimal, and preseason temperature continued to be the most important driver of both SOS_15_ and SOS_50_ (Fig. S21).

### Environmental predictors of spatial variation in carry-over effects

**Figure 5.**
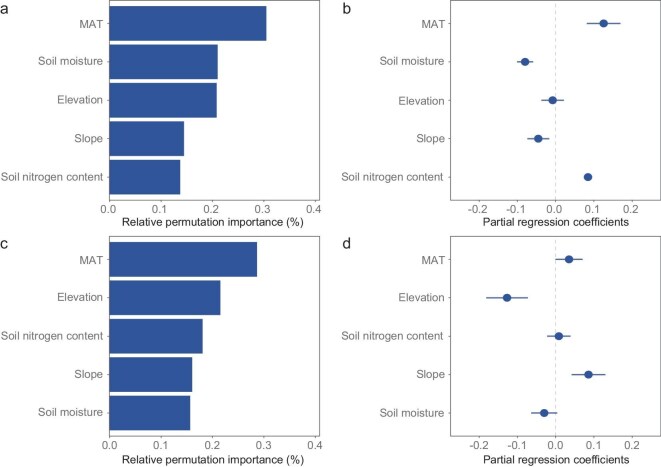
Environmental predictors of spatial variation in phenological carry-over effects on leaf senescence and leaf-out. (a and c) Relative permutation importance of five environmental predictors in random forest models, ranked by their contribution to explaining spatial variation in the SOS–EOS effect (a) and EOS–SOS effect (c). The SOS–EOS effect quantifies the influence of the SOS (SOS_15_) on the onset of leaf senescence (EOS_10_), while the EOS–SOS effect captures the influence of EOS_10_ on the following SOS_15_. (b and d) Bootstrapped partial regression coefficients (mean ± SD) showing the linear influence of each predictor on the SOS–EOS (b) and EOS–SOS (d) effects. Coefficients were averaged across 100 multivariate linear models. All variables were standardized to allow direct comparison of effect sizes. Predictors include MAT, soil moisture, elevation, soil nitrogen content and slope.

To determine the environmental drivers of spatial variation in the carry-over effects, we used random forest models to quantify the influence of climate, topography and soil factors on pixel-level SOS–EOS and EOS–SOS relationships [47]. Five predictor variables were included in the random forest models: mean annual temperature (MAT), soil moisture, slope, elevation and soil nitrogen content. Based on the models (*R*^2^ = 0.19 for the SOS–EOS effect and 0.35 for the EOS–SOS effect), MAT emerged as the most important predictor of spatial variation in both the SOS–EOS and EOS–SOS effects (Fig. 5a and c). Specifically, higher MAT was associated with stronger SOS–EOS relationships, while increased soil moisture tended to weaken this effect (Fig. 5b). Conversely, the EOS–SOS effect was more pronounced in cold regions, although the direction varied across sites and the overall effect was small (Fig. 5d). These findings align with the observed spatial distribution of dominant carry-over effects (Fig. 2e). Similar patterns were found when testing different combinations of SOS and EOS stages (SOS_15_ combined with EOS_50_, SOS_50_ combined with EOS_10_, and SOS_50_ combined with EOS_50_; Figs S22–S24).

## DISCUSSION

Our study quantified phenological carry-over effects between the start and end of the growing season in the world’s deciduous forests, savannas, shrublands and grasslands. We found a widespread positive SOS–EOS effect (81% of pixels, 29% significant), as earlier leaf-out tends to advance leaf senescence across the majority of deciduous vegetation. By contrast, the EOS–SOS effect was primarily pronounced in high-latitude regions and varied in direction across pixels. Our findings revealed a dampening interaction between the two carry-over effects, whereby a stronger influence of one tended to weaken the other (Fig. 1d and e). This trade-off resulted in a spatial pattern in which sites with a strong EOS–SOS effect tended to exhibit a weaker SOS–EOS effect, and vice versa (Fig. 3a). Temperature and water availability emerged as the key environmental drivers modulating the strength and direction of carry-over effects (Fig. 5). Specifically, higher MAT was associated with stronger SOS–EOS relationships globally, whereas increased soil moisture tended to weaken these effects. Conversely, the EOS–SOS effect was more pronounced in cold regions. Notably, the SOS exerted a stronger influence on the EOS than radiation and precipitation, and often even exceeded the effect of temperature across vegetation types in the satellite data, while spring temperature remained the most important driver of the SOS across all regions. This underscores the central role of internal physiological and growth constraints and highlights the need to integrate carry-over effects into phenological models to improve predictions of vegetation seasonality and carbon cycles.

### SOS–EOS effect

Several hypotheses may explain the widespread carry-over effect of the SOS on the EOS observed in our study. An earlier SOS advances the onset of photosynthesis, potentially accelerating development and growth in the early growing season [6,11]. In temperate trees, this accelerated development has been shown to drive an earlier onset of leaf senescence [23,24,48]. Indeed, this effect was particularly evident in temperate deciduous forests, where both experimental and observational data have demonstrated the influence of developmental speed on EOS [23,48–50]. Our finding that preseason temperature advances EOS_10_ is consistent with this mechanism, as warmer conditions tend to enhance vegetation activity in temperate regions, provided that water is not severely limiting [48].

Accelerated early-season development may also influence the EOS through internal feedbacks related to plant nutrient balance, especially nitrogen and non-structural carbohydrate (NSC) storage [51–53]. In herbaceous plants, experimental studies have shown that early-season productivity can advance senescence through source-sink feedbacks [51,53]. Consistent with this, we observed strong SOS–EOS effects in grasslands. Earlier vegetation greening may also increase vulnerability to summer drought [29,54], which should be a selective factor in regions where water is limiting [55]. Our results support this expectation, showing that the carry-over effect of the SOS on the EOS intensifies in regions with low soil moisture, such as the dry and continental grasslands of Central North America and West Asia. Further research is needed to untangle the relative contributions of source–sink feedback and water limitation in driving EOS dynamics in these dryland systems.

Leaf lifespan could be another contributing factor to the observed SOS–EOS linkage [22,25,56]. Leaf aging is partly driven by the accumulation of reactive oxygen species (ROS), and earlier leaf-out may accelerate ROS-related aging due to increased exposure to solar radiation [57–59]. However, across temperate and boreal regions, solar radiation had a relatively minor effect on the EOS timing compared to the SOS and temperature. Moreover, if leaf age was the dominant control on senescence, one would expect stronger SOS effects on later senescence stages (EOS_50_). However, we observed the opposite; the SOS had a stronger influence on the onset of senescence (EOS_10_), while EOS_50_ was more responsive to late-season temperature. Thus, while leaf aging may contribute to senescence onset, it is late-season environmental conditions—particularly temperature—that determine the progression of senescence [48,60].

Overall, our results support a two-phase process of senescence in temperate and boreal regions. Its onset is shaped by early-season developmental cues and drought stress [28,48,59], while its progression is modulated by late-season climate. By contrast, in tropical savannas and grasslands, increased radiation and reduced precipitation were associated with an earlier EOS, while temperature showed no significant effect.

### EOS–SOS effect

The strongest effects of the EOS on the subsequent SOS were found in boreal regions, where plants experience long dormancy periods [11,61]. However, the effect direction varied across pixels, with a delayed EOS sometimes delaying and at other times advancing the SOS [39]. In continental areas with cold winters, a positive EOS–SOS relationship may reflect dormancy and chilling requirements [36,37]; a later EOS is associated with a later bud set, which can postpone dormancy induction, reduce chilling exposure, and thereby delay spring budburst [14,20,38]. The stronger effect of the EOS on SOS_15_ in high-latitude regions supports this expectation [62]. Many species from temperate and cold regions exhibit pronounced chilling requirements [20,63,64]. In these colder climates, inter-annual variation in chilling exposure may depend more on the timing of dormancy onset than on absolute winter temperatures, which are generally well below upper chilling thresholds [65]. EOS timing may to some extent reflect the timing of dormancy induction due to shared temperature dependence [4,66], potentially explaining the observed association between EOS and the following SOS. Conversely, a late EOS could advance the SOS if associated with reduced dormancy depth [39]. Additional factors such as internal carbohydrate and nutrient availability for spring development [62,67,68] may also influence the EOS–SOS relationship. For instance, delayed leaf senescence is generally associated with increased nutrient resorption, facilitating leaf emergence in the following year [40,41]. Nevertheless, despite the EOS–SOS effects in high-latitude regions, spring temperature remained the most important and consistent driver of the SOS, especially when using SOS_50_ instead of SOS_15_, highlighting its central role in shaping future phenological responses [38].

We also found evidence of an EOS–SOS effect in grasslands and savannas, where phenological carry-over processes have received little attention [15,16]. Although chilling accumulation can be important for dormancy release in some herbaceous species in temperate and boreal regions, most herbaceous species employ different dormancy and regeneration strategies compared to long-lived woody plants [69]. Here, EOS timing may affect the SOS through legacy effects on reproductive development, nutrient remobilization and belowground carbohydrate storage [70,71], as well as through seed maturation and dispersal [72]. Nevertheless, the EOS effect on the SOS was generally weak, likely because grassland phenology is strongly governed by spring temperature in temperate and boreal climates, and water availability [73,74] in tropical regions, which may override prior-season legacies. Experimental studies across diverse plant systems are needed to clarify the physiological pathways linking the EOS to the subsequent SOS.

### Relationships between SOS and EOS carry-over effects

We discovered a dampening interaction between the two carry-over effects, where the presence of one effect reduces the impact of the other (Fig. 3a). Aligning with this, only one carry-over effect was significant in most pixels (‘+0’, ‘0−’, ‘0+’ and ‘−0’ categories; Fig. 3b). Across the study area, 27% of pixels showed a significant SOS–EOS effect only (26% positive; 1% negative), 5% showed a significant EOS–SOS effect only, and just 2% exhibited significant effects in both relationships. SOS–EOS effects tend to prevail in temperate regions, where growth-stress trade-offs during the growing season amplify the influence of spring phenology on autumn senescence. In contrast, EOS–SOS effects dominate in high-latitude boreal regions, where autumnal dormancy regulation and chilling constraints may exert stronger control over subsequent spring leaf-out. These phenological constraints likely help plants balance the competing demands of growth maximization and stress avoidance, thereby preventing shifts in seasonal timing that would place key life stages out of sync with favorable environmental conditions.

### Implications

Our results highlight that internal physiological regulation plays a critical role in shaping ecosystem functioning and mediating ecosystem responses to climate change [75–77]. Although rising temperatures are expected to advance spring phenology and slow the progression of autumn leaf senescence [4,7], the observed carry-over effects we identify may dampen the net phenological response. In particular, the widespread and consistent advancing effects of an earlier SOS on the EOS suggest that further SOS advances may counteract autumn warming-driven delays in leaf senescence. While we filtered low-quality satellite data and validated our results using ground observations, phenology metrics derived from greenness amplitude can remain uncertain, especially in tropical and subtropical regions where seasonal greenness signals are weak. Targeted, climate-controlled experiments will be essential to further resolve the mechanisms underpinning any carry-over processes across biogeographic regions.

Our findings also have important consequences for phenological and ecosystem modeling. Most current land-surface and dynamic global vegetation models rely on externally driven temperature thresholds or fixed leaf-longevity assumptions [78], largely neglecting internal carry-over processes between phenophases. Our results suggest that accounting for these internal constraints is essential for reliable projections of growing-season dynamics and carbon–climate feedback. Incorporating empirically derived carry-over relationships—particularly the dominant influence of spring phenology on autumn senescence—can improve phenology modules and model accuracy [16,23], enhancing our capacity to forecast ecosystem responses of seasonal vegetation to environmental change.

## METHODS

### Satellite-derived phenology dataset

The start (SOS) and end (EOS) of the growing season across the world’s seasonal vegetation from 2001 to 2022 were extracted from the MODIS Global Vegetation Phenology product [42]. This product provides phenological metrics at a spatial resolution of 500 m for every vegetated pixel on land, derived from time series of the 2-band EVI (EVI2; Fig. S1). The SOS was estimated as the date when the greenness index first increased by >15% (Greenup, SOS_15_) and 50% (MidGreenup, SOS_50_) of the seasonal amplitude. The EOS was defined as the date when greenness had dropped by 10% (Senescence, EOS_10_) and 50% (MidGreendown, EOS_50_) to represent the onset of senescence and mid-senescence, respectively. To capture the late phase of leaf senescence, we additionally extracted the date when greenness had dropped by 85% (Dormancy, EOS_85_). We resampled the MODIS phenology data to a 0.25° resolution by calculating the median phenological date of all 500 m pixels within each 0.25° grid cell. To ensure data quality, we excluded pixels labeled as ‘poor’ in the product quality assessment and removed observations deviating by more than 3.5 times the median absolute deviation for each pixel. For each pixel, only years with available records of current-year SOS and EOS as well as previous-year EOS were retained, and pixels with fewer than 15 years of phenological data were excluded. After filtering, approximately 56% of pixels were retained.

### Climate geography dataset

Daily air temperature, precipitation and net short-wave radiation data at 0.25° resolution were obtained from the Global Land Data Assimilation System (GLDAS v2.1) [79]. The 30-year mean annual temperature data were sourced from Climatologies at High resolution for the Earth Land Surface Areas (CHELSA) V2.1 at a resolution of 30′′ [42]. Topography data, including elevation and slope, were derived from EarthEnv (https://www.earthenv.org/topography), which utilizes the digital elevation model products of the global 250 m GMTED2010 and near-global 90 m SRTM4.1dev [43]. Soil moisture data were obtained from 10 km resolution maps sourced from GLDAS2.0 [79], ERA5 [80] and MERRA2 [81]. Soil nutrient information was sourced from the WISE30sec [82] database and SoilGrids [83]. These datasets were all resampled to 0.25° using the median value to align with the resolution of the phenology data.

### Vegetation classification dataset

The vegetation classification dataset was derived from the MODIS Land Cover Type (MCD12Q1) Version 6.1 data product (https://lpdaac.usgs.gov/products/mcd12q2v061/). This product provides global land cover types at yearly intervals from 2001 to 2020, with 2020 used as the reference year in this study. We excluded evergreen vegetation and selected eight distinct seasonal vegetation types: deciduous needleleaf forests; deciduous broadleaf forests; mixed forests; closed shrublands; open shrublands; woody savannas; savannas; and grasslands. We used Terrestrial Ecosystems of the World from WWF-US to classify boreal, temperate and tropical regions [84]. Tropical regions include pixels classified as Tropical & Subtropical Moist Broadleaf Forests, Tropical & Subtropical Dry Broadleaf Forests, Tropical & Subtropical Coniferous Forests, Tropical & Subtropical Grasslands, Savannas & Shrublands, Deserts & Xeric Shrublands, and Mangroves. Temperate regions encompass Temperate Broadleaf & Mixed Forests, Temperate Conifer Forests, Temperate Grasslands, Savannas & Shrublands, Montane Grasslands & Shrublands, Mediterranean Forests, and Woodlands & Scrub. Boreal regions are defined as pixels classified under Boreal Forests/Taiga, Tundra, and Flooded Grasslands & Savannas.

Boreal deciduous needleleaf forests, deciduous broadleaf forests, mixed forests and woody savannas were merged into boreal forests. Boreal closed shrublands, open shrublands and grasslands were merged into boreal shrublands. Temperate deciduous needleleaf forests and deciduous broadleaf forests were combined into temperate deciduous forests. Temperate closed shrublands, open shrublands, woody savannas and savannas were merged into temperate grasslands. Tropical deciduous needleleaf forests, deciduous broadleaf forests, mixed forests and woody savannas were merged into tropical woody savannas. Tropical savannas, closed shrublands and open shrublands were combined into tropical grasslands. Ultimately, we distinguished eight vegetation types in our analyses (Fig. S3): boreal forest; boreal savanna; boreal shrubland; temperate deciduous forest; temperate mixed forest; temperate grassland; tropical savanna; and tropical grassland.

### Ground observations

Ground phenological observations were obtained from the PEP network (http://www.pep725.eu/), which provides records of phenological events across Central Europe from 1950 to 2023 [85]. The dates of leaf-out and leaf senescence of each tree were defined based on the Biologische Bundesanstalt, Bundessortenamt und Chemische Industrie (BBCH) codes 13 and 94, respectively, which was represented by day of the year (DOY) [85,86]. BBCH 13 refers to the date when 50% of a tree’s leaves have unfolded, while BBCH 94 indicates the date when 50% of the leaves show autumnal coloring. To avoid abnormal dates resulting from measurement error or extreme climate, leaf-out dates later than DOY 180 and leaf senescence dates earlier than DOY 180 were excluded from the analysis. Additionally, potential outliers were removed by excluding observations that deviated by more than 3.5 times the median absolute deviation for each site–species combination. For each site and species, only years with available records of current-year leaf-out and leaf senescence, as well as next-year leaf-out, were retained. Site-species combinations with fewer than 20 years of phenological data were excluded from the analysis. In total, 2994 phenological sites and 321 639 phenological observations were selected for four dominant deciduous tree species: *Aesculus hippocastanum, Fagus sylvatica, Betula pendula* and *Quercus robur*. We extracted satellite-derived forest phenology data from the main PEP distribution regions, including Germany, Austria, Switzerland, Czechia, Poland, Slovakia and Hungary, and compared leaf-out and leaf senescence dates, as well as the corresponding carry-over effects, between satellite-derived and ground-based observations. Environmental data (i.e. temperature, radiation and precipitation) matching the PEP network coordinates were extracted from the E-OBS gridded dataset (https://www.ecad.eu/dailydata/index.php), spanning the same period from 1950 to 2023.

### Statistical analysis

To determine the optimal preseason of each climate variable for each phenophase, while excluding the influences of year and non-target climate variables, we ran a multilinear regression model with the target phenophase as the response variable and the previous phenophase, temperature, precipitation, radiation and year as explanatory variables. This model was run 10 times per pixel, each using climate variable averaged over different time windows ranging from 15 to 150 days before the multi-year average phenophase date, with steps of 15 days (i.e. 0–15, 0–30, 0–45, …, 0–150 days). The optimal preseason length for each climate variable at each pixel was defined as the period before the multi-year average phenophase date with the smallest *P* value for the coefficient between phenophase and climate variable [63,87]. We then calculated the average temperature, total precipitation and radiation during the optimal preseason for each year at each pixel.

To investigate the effects of the SOS on the EOS (SOS–EOS effect) and the EOS on the subsequent SOS (EOS–SOS effect), we conducted multilinear regression models, considering preseason temperature and year as covariates. To compare the relative effects of the two carry-over effects, we determined the dominant carry-over effect using their absolute coefficients (SOS–EOS minus EOS–SOS). To reveal the phenological carry-over types, we combined the two-carry-over effects at each pixel and classified them into eight carry-over types based on their effect direction and significance level at *P *< 0.05. For example, ‘++’ indicates a positive SOS–EOS effect and a positive EOS–SOS effect, ‘+0’ signifies a positive SOS–EOS effect with no significant EOS–SOS effect, and ‘+−’ represents a positive SOS–EOS effect and a negative EOS–SOS effect.

To quantify the relative impacts of carry-over effects versus environmental cues on subsequent phenological events, we standardized all variables and implemented Bayesian linear mixed-effects models using the ‘brms’ package in R [45,88]. The EOS (or SOS) was modeled as the response variable, with the SOS (or previous EOS), preseason temperature, precipitation and radiation as fixed predictors. To account for spatial-temporal dependence, we specified pixel identity as a random intercept and allowed year to vary as a random slope within each pixel [formula: X ∼ predictors + (year||pixel)]. This structure accounts for baseline differences among pixels and for site-specific temporal trends in phenology. Models were fitted separately for each vegetation type, with weakly informative priors and four chains of 2000 iterations each, using the CmdStanR backend for efficient sampling. Posterior mean standardized coefficients (β) were extracted to assess and compare the relative strength of carry-over versus climatic effects on phenological timing. To identify the most important predictor of the EOS or SOS, we additionally fitted multiple linear regression models with the same set of predictors, selecting the variable with the lowest *P* value as the strongest predictor. Parallel analyses were conducted for long-term ground-based leaf-out and senescence observations.

To assess the factors driving spatial variation in the carry-over effects, we quantified the impact of climate and topography factors on SOS–EOS and EOS–SOS effects, respectively, by applying random forest models [47]. Five predictor variables, including pixel-level MAT, soil moisture, soil nitrogen content, slope and elevation, were included after evaluating multicollinearity among variables using variance inflation factors [47,89]. We quantified the relative permutation importance of the selected variables using random forest models implemented with the ‘ranger’ R package [90], using 500 trees and default parameter settings. To examine the effect of the selected predictors on the two carry-over effects, we fitted a multivariate regression model and calculated the corresponding regression coefficients. Bootstrapped partial regression coefficients for each variable were calculated by averaging the partial regression coefficients from 100 multivariate linear models. All response and predictor variables were standardized to enable direct comparison of effect sizes.

## Supplementary Material

nwag082_Supplemental_File

## Data Availability

Satellite-derived phenology data were extracted from the MODIS Global Vegetation Phenology product (https://doi.org/10.5067/MODIS/MCD12Q2.061). Daily climate data were obtained from the Global Land Data Assimilation System (GLDAS v2.1; https://disc.gsfc.nasa.gov/datasets). Thirty-year mean annual temperature data were sourced from CHELSA v2.1 (https://www.chelsa-climate.org/models/chelsa). Topographic data were derived from EarthEnv (https://www.earthenv.org/topography). Soil moisture was obtained from GLDAS v2.0 (https://disc.gsfc.nasa.gov/datasets), ERA5 (https://cds.climate.copernicus.eu/datasets) and MERRA-2 (https://gmao.gsfc.nasa.gov/gmao-products/merra-2/). Soil nutrient information was sourced from WISE30sec (https://data.isric.org/) and SoilGrids (https://soilgrids.org/). Vegetation data were derived from the MODIS Land Cover Type product (MCD12Q1 v6.1; https://lpdaac.usgs.gov/products/mcd12q1v061/). Ecoregion data were obtained from the Terrestrial Ecoregions of the World dataset provided by WWF-US (https://2c1forest.databasin.org/). Ground-based phenological observations were obtained from the Pan European Phenology (PEP) network (http://www.pep725.eu/). Environmental data corresponding to the PEP phenological observations were extracted from the E-OBS gridded dataset (https://www.ecad.eu/dailydata/index.php).
