# Supplementary material for: Carry-over effects between spring and autumn phenology differ among the world’s biomes
Source: Natl Sci Rev. 2026 Feb 6;13(5):nwag082. doi: 10.1093/nsr/nwag082 (PMC12993445; doi:10.1093/nsr/nwag082)
Supplement: nwag082_Supplemental_File [file nwag082_supplemental_file.docx]

**Supplementary Information for**

**Carry-over effects between spring and autumn phenology differ among the World’s biomes**

**Authors:** Zhaofei Wu^1,2^, Yongshuo H. Fu^1,3*^, Thomas W. Crowther^4,5^, Susanne S. Renner^6^, Yann Vitasse^2,7^, Lidong Mo^8^, Yibiao Zou^2,9^, Leila Mirzagholi^10^, Mingwei Li^1^, Dominic Rebindaine^2,9^, Yufeng Gong^1^, Zhendong Guo^1^, Nan Wang^1^, Constantin M. Zohner^4,9*^

**Affiliations:**

^1^College of Water Sciences, Beijing Normal University, Beijing 100875, China;

^2^Ecosystem Ecology, Swiss Federal Institute for Forest, Snow and Landscape Research (WSL), Birmensdorf, 8903, Switzerland;

^3^Department of Biology, University of Antwerp, Antwerpen, 2000, Belgium;

^4^BRANCH Institute, Zug, 6300, Switzerland;

^5^Environmental Science and Engineering, King Abdullah University of Science and Technology (KAUST), Thuwal, 23955, Saudi Arabia;

^6^Department of Biology, Washington University in St. Louis, Saint Louis, MO 63130, USA;

^7^Oeschger Centre for Climate Change Research, University of Bern, Bern, 3012, Switzerland;

^8^College of Life Science, Nankai University, Tianjin, 300071, China;

^9^Institute of Integrative Biology, ETH Zurich, Zurich, 8092, Switzerland;

^10^Department of Civil and Environmental Engineering, Massachusetts Institute of Technology, Cambridge, MA 02139, USA.

* Authors for correspondence: Constantin M. Zohner (constantin.zohner@usys.ethz.ch) and Yongshuo H. Fu (yfu@bnu.edu.cn)

**This file includes:**

Table S1

Figures S1 to S24

**Table. S1.** Summary of evidence for carry-over effects between spring leaf-out (SOS) and autumn senescence (EOS) across regions and data sources. SOS–EOS and EOS–SOS represent the effects of SOS on EOS and EOS on SOS, respectively; “P” and “N” indicate positive and negative carry-over effects. R indicates the correlation or the standardized effect.

| **Data source** | **Regions**  **(time range)** | **SOS-EOS** | **EOS-SOS** | **References** |
| --- | --- | --- | --- | --- |
| Satellite-derived observation | Northern Hemisphere (1982-1999) | R=0.55 | NA | Zhang et al. 2024 |
|  | Northern Hemisphere (2000-2016) | R=0.48 | NA |  |
|  | Northern Hemisphere (1982-2018) | R=0.41~0.45 | NA | Yuan et al. 2024 |
|  | Northern Hemisphere (1982-2022) | 0.15 days/year | Roughly half were positive and half negative | Tang et al. 2025 |
|  | Southern Hemisphere (1982-2022) | 0.18 days/year | -0.05 days/year |  |
|  | Boreal region (1982-2015) | NA | 0.5~1 days/day | Shen et al. 2020 |
|  | Temperate Ecosystems (1982-2015) | NA | Weak negative correlation |  |
|  | Northern Hemisphere (1982-2011) | P: 60%, N: 40% | NA | Liu et al. 2016 |
|  | American (2000-2012) | 1.1 days/day | NA | Keenan et al. 2015 |
| Ground observation | Europe (1963-2015) | 0.17 days/year | NA | Tang et al. 2025 |
|  | American (1989-2012) | 0.52~0.66 days/day | NA | Keenan et al. 2015 |
|  | Europe (1950-2011) | R=0.1 | NA | Fu et al. 2014 |
| Experiment | Europe (2009-2011) | 0.36~0.47 days/day | NA | Fu et al. 2014 |


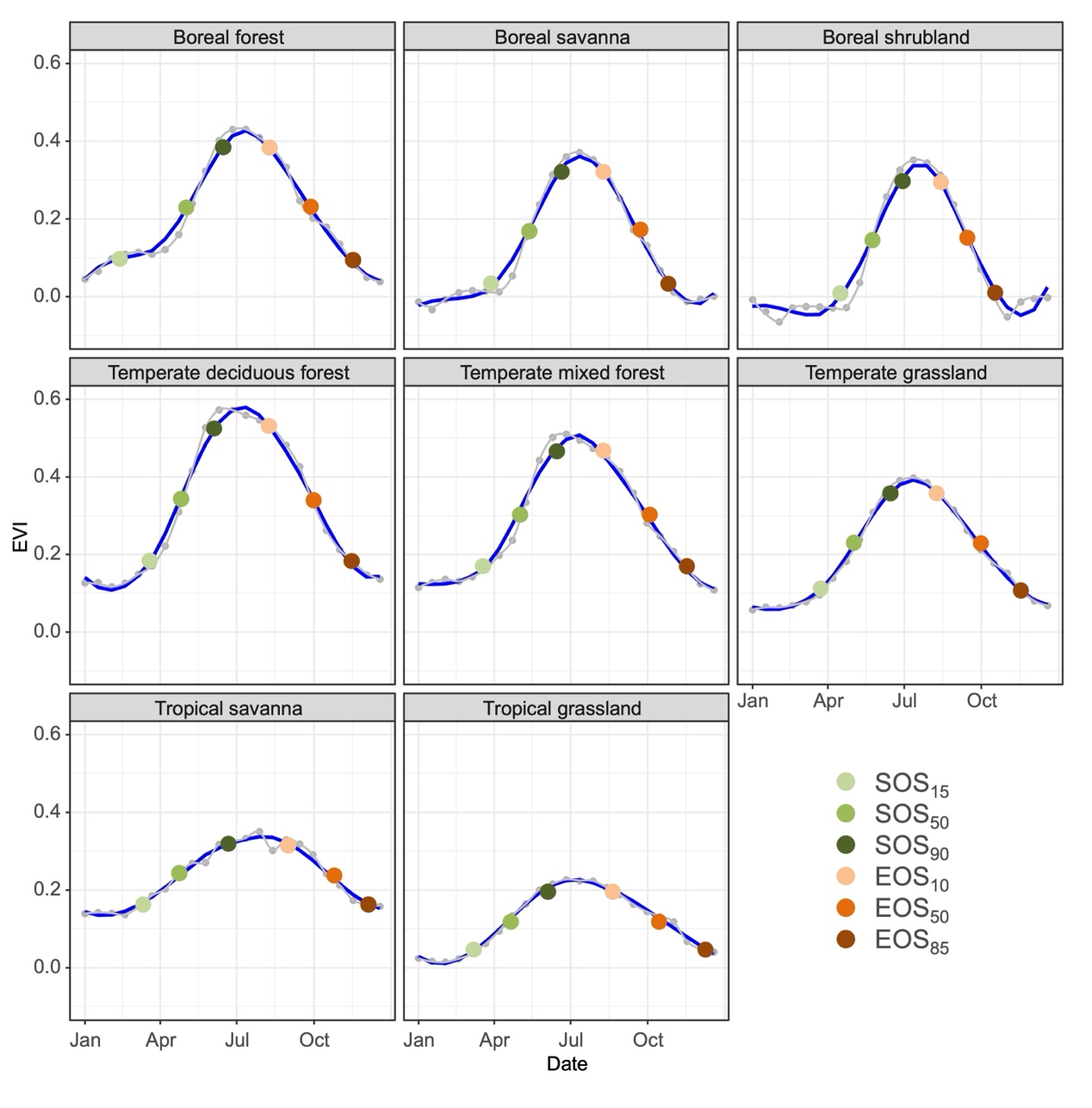


**Fig. S1. Average seasonal dynamics of vegetation greenness across vegetation types.** Mean seasonal trajectories of the Enhanced Vegetation Index (EVI) are shown for major vegetation types in the Northern Hemisphere. Gray points and lines represent the observed EVI values, while blue lines show Savitzky–Golay–smoothed fitted time series. Colored points indicate key phenological transition dates identified using relative amplitude thresholds. For example, SOS_15_ was estimated as the date when the greenness index first increased by more than 15% of the seasonal maximum, and EOS_10_ was defined as the date when greenness had declined to 10% of the seasonal maximum.


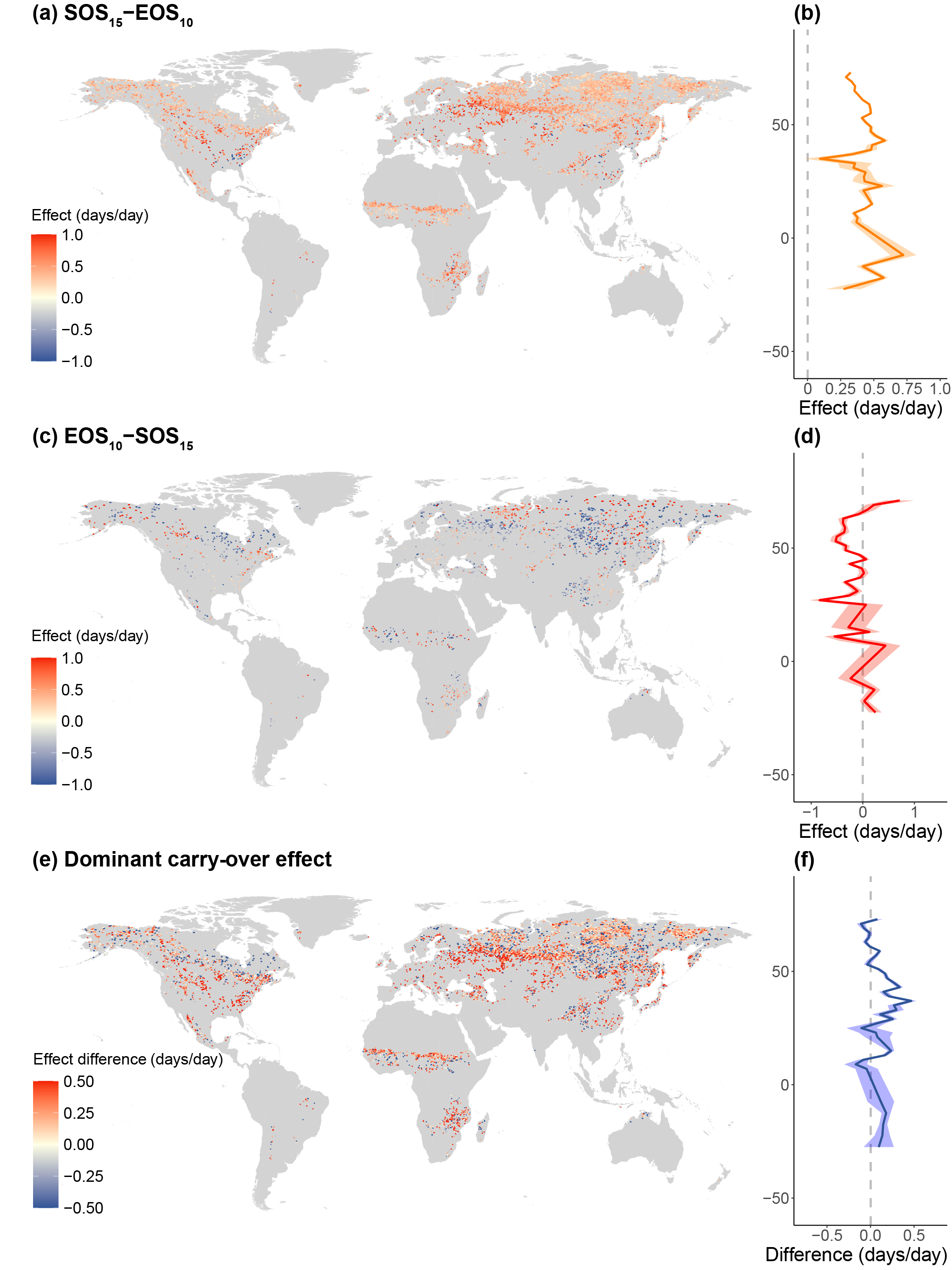


**Fig. S2. Global carry-over effects between leaf-out and leaf senescence for the significant pixels (*p* < 0.05). a**, Map showing the effect of leaf-out onset (SOS_15_) on leaf senescence onset (EOS_10_) [SOS-EOS effect] at 0.25° resolution, derived from multilinear regression models with year and preseason temperature as covariates. **b**, Latitudinal variation in the SOS-EOS effect, with solid lines representing mean regression coefficients and shaded areas indicating standard deviations, summarized for each 2° latitude band (bands with fewer than 100 pixels were removed). **c**, **d**, Map and latitudinal variation for the effect of EOS_10_ on SOS_15_ (EOS-SOS effect). **e**, Map illustrating the dominant carry-over effect, calculated as the difference between the absolute coefficients of the SOS-EOS and EOS-SOS effects. Red pixels indicate regions where SOS-EOS effect dominates, while blue pixels indicate stronger EOS-SOS effect. **f**, Latitudinal variation in the relative importance of SOS-EOS versus EOS-SOS effects, with positive values reflecting stronger SOS-EOS effect and negative values indicating stronger EOS-SOS effect.


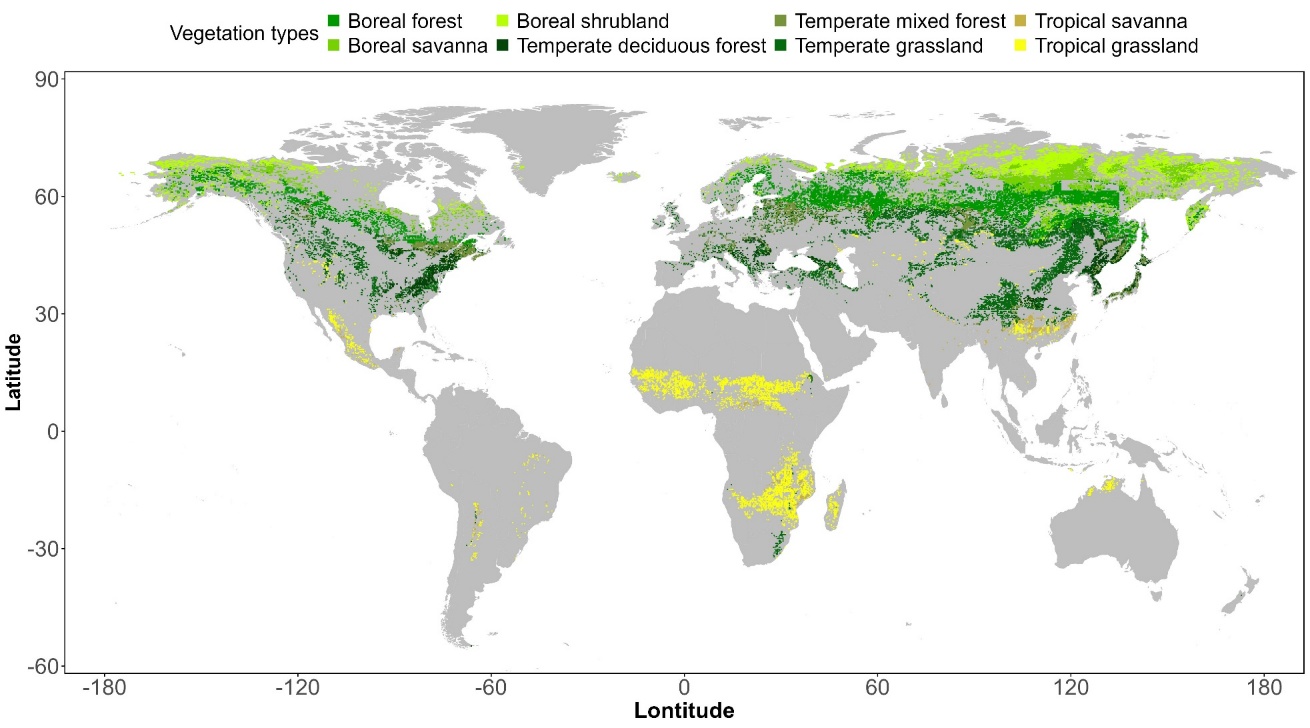


**Fig. S3. Distribution of the eight vegetation types studied here.** Vegetation types are based on the MODIS land cover product (MCD12Q1 Version 6.1, https://lpdaac.usgs.gov/products/mcd12q2v061/). Climate zones (tropical, temperate, boreal) are based on the Terrestrial Ecosystems of the World from WWF-US (https://www.worldwildlife.org/publications/terrestrial-ecoregions-of-the-world, see Methods).


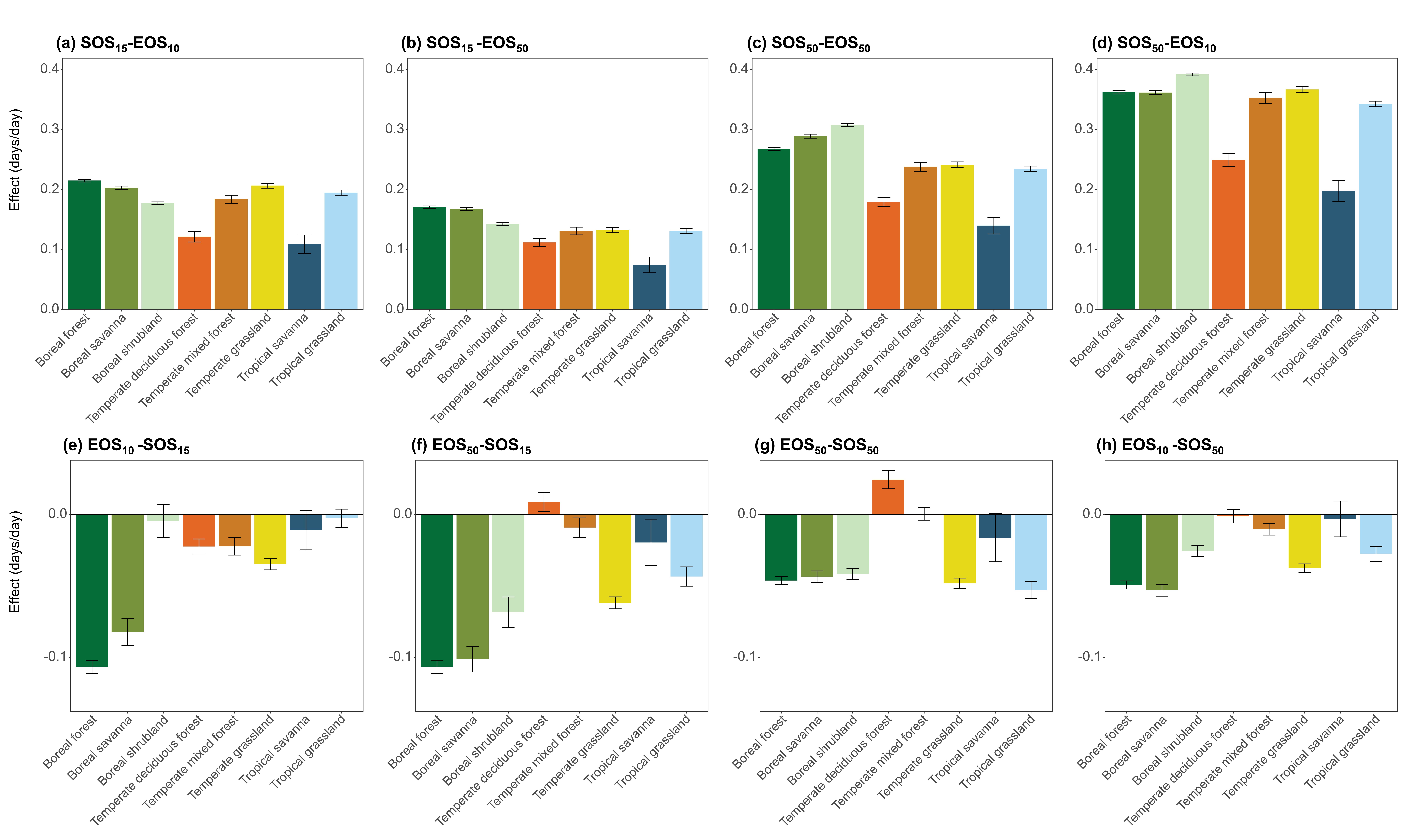


**Fig. S4. Phenological carry-over effects across vegetation types.** Each panel shows the phenological carry-over effects in terms of days per day for different vegetation types. **a**, SOS_15_-EOS_10_: Effect of leaf-out onset (SOS_15_) on leaf senescence onset (EOS_10_). **e**, EOS_10_-SOS_15_: Effect of EOS_10_ on the following SOS_15_. **b**, SOS_15_-EOS_50_: Effect of SOS_15_ on mid-senescence (EOS_50_). **f**, EOS_50_-SOS_15_: Effect of EOS_50_ on the following SOS_15_. **c**, SOS_50_-EOS_50_: Effect of mid leaf-out (SOS_50_) on EOS_50_. **g**, EOS_50_-SOS_50_: Effect of EOS_50_ on the following SOS_50_. **d**, SOS_50_-EOS_10_: Effect of SOS_50_ on EOS_10_. **h**, EOS_10_-SOS_50_: Effect of EOS_10_ on the following SOS_50_. Error bars represent standard errors.


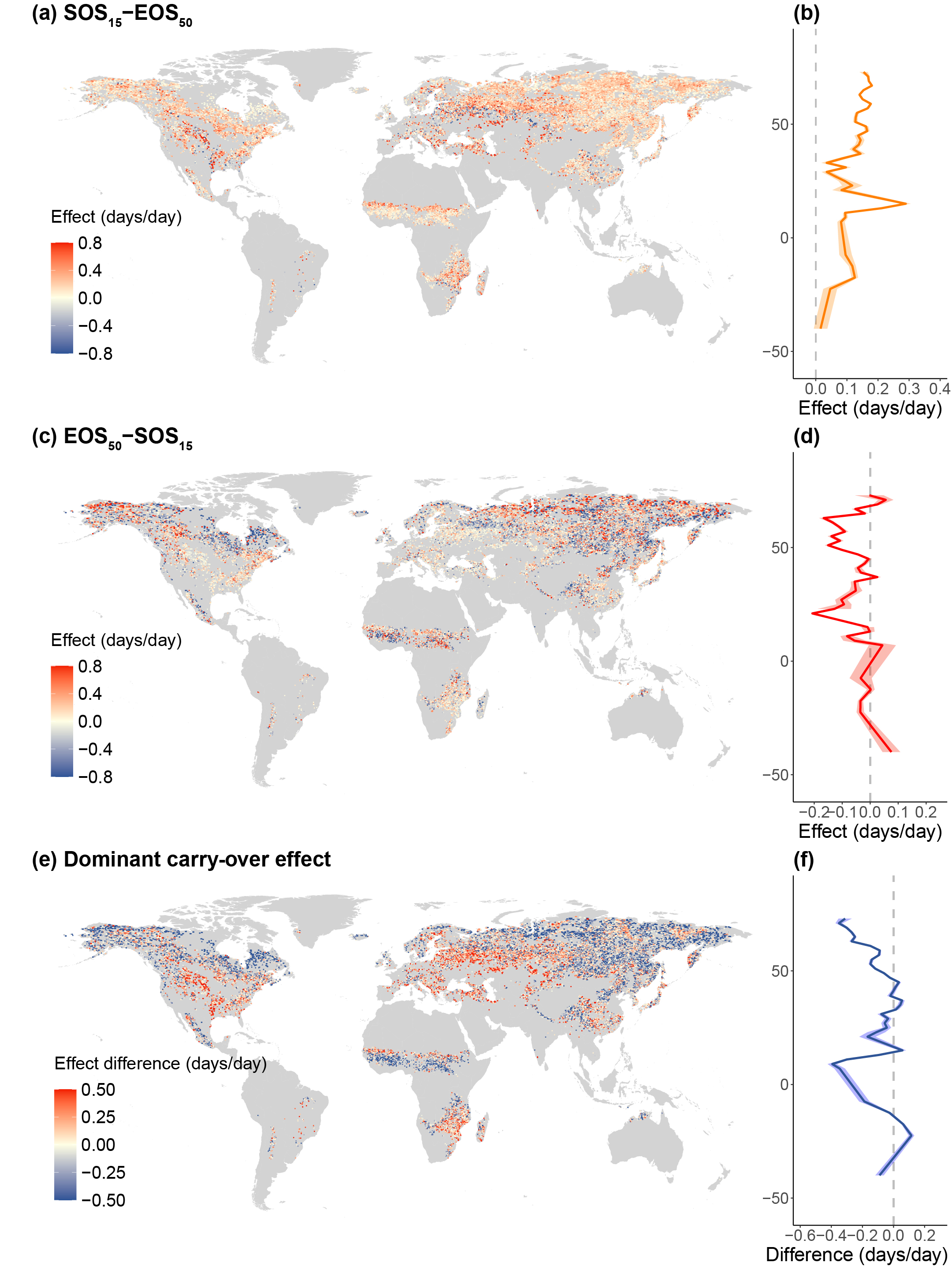


**Fig. S5. Global carry-over effects between leaf-out onset (SOS_15_) and mid-senescence (EOS_50_). a**, Map showing the effect of SOS_15_ on EOS_50_ [SOS-EOS effect] at a 0.25° resolution, derived from multilinear regression models with year and preseason temperature as covariates. **b**, Latitudinal variations in the SOS-EOS effect, with solid lines representing mean regression coefficients and shaded areas indicating standard deviations, summarized for each 2° latitude band (bands with fewer than 100 pixels were removed). **c**, **d**, Map and latitudinal variation for the effect of EOS_50_ on subsequent SOS_15_ (EOS-SOS effect). **e**, Map illustrating the dominant carry-over effect, calculated as the difference between the absolute coefficients of the SOS-EOS and EOS-SOS effects. Red pixels indicate regions where SOS-EOS effects dominate, while blue pixels indicate stronger EOS-SOS effects. **f**, Latitudinal variations in the relative importance of SOS-EOS versus EOS-SOS effects, with positive values reflecting stronger SOS-EOS effects and negative values indicating stronger EOS-SOS effects.


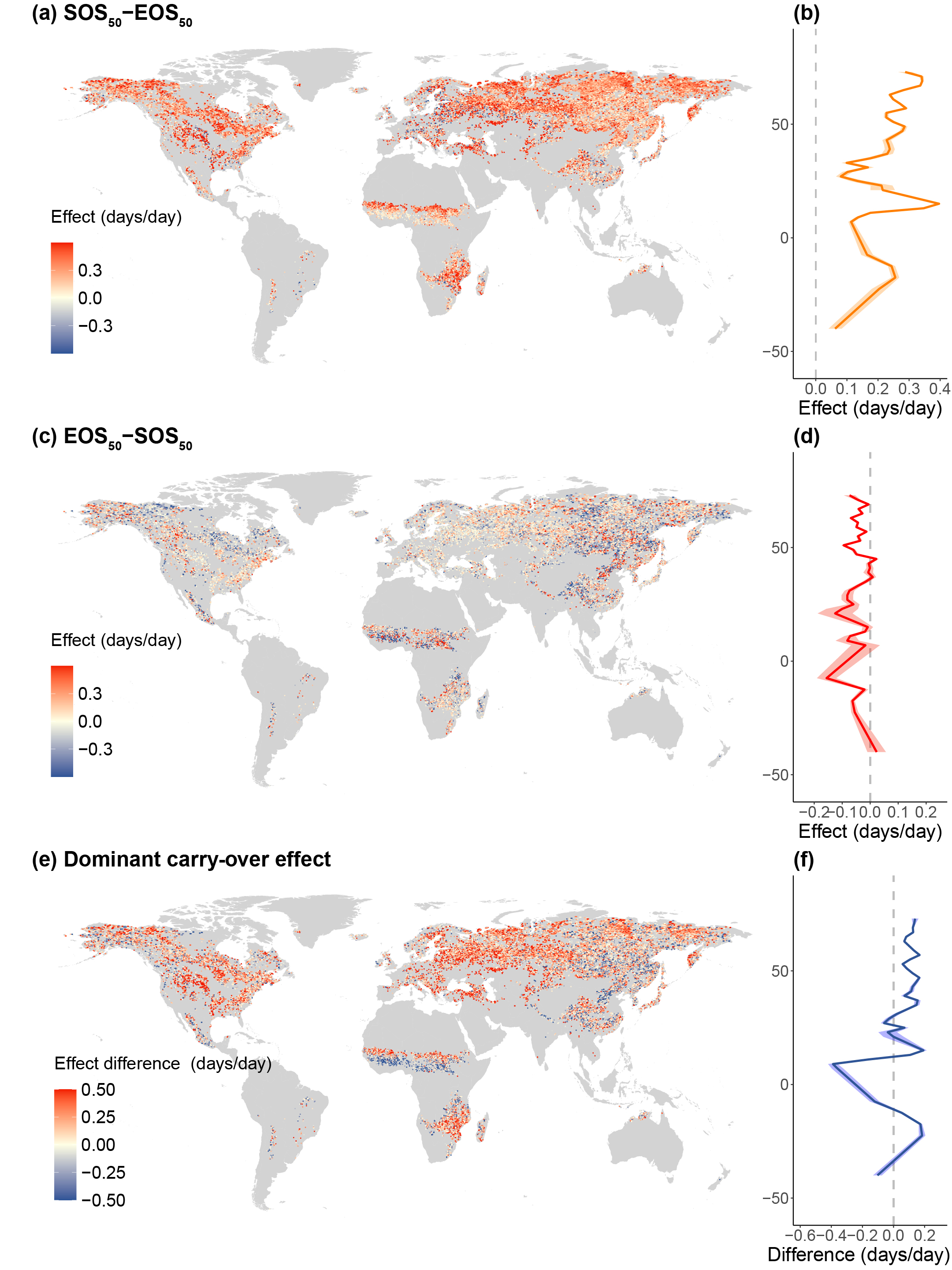


**Fig. S6. Global carry-over effects between mid leaf-out (SOS_50_) and mid-senescence (EOS_50_). a**, Map showing the effect of SOS_50_ on EOS_50_ [SOS-EOS effect] at a 0.25° resolution, derived from multilinear regression models with year and preseason temperature as covariates. **b**, Latitudinal variations in the SOS-EOS effect, with solid lines representing mean regression coefficients and shaded areas indicating standard deviations, summarized for each 2° latitude band (bands with fewer than 100 pixels were removed). **c**, **d**, Map and latitudinal variation for the effect of EOS_50_ on subsequent SOS_50_ (EOS-SOS effect). **e**, Map illustrating the dominant carry-over effect, calculated as the difference between the absolute coefficients of the SOS-EOS and EOS-SOS effects. Red pixels indicate regions where SOS-EOS effects dominate, while blue pixels indicate stronger EOS-SOS effects. **f**, Latitudinal variations in the relative importance of SOS-EOS versus EOS-SOS effects, with positive values reflecting stronger SOS-EOS effects and negative values indicating stronger EOS-SOS effects.


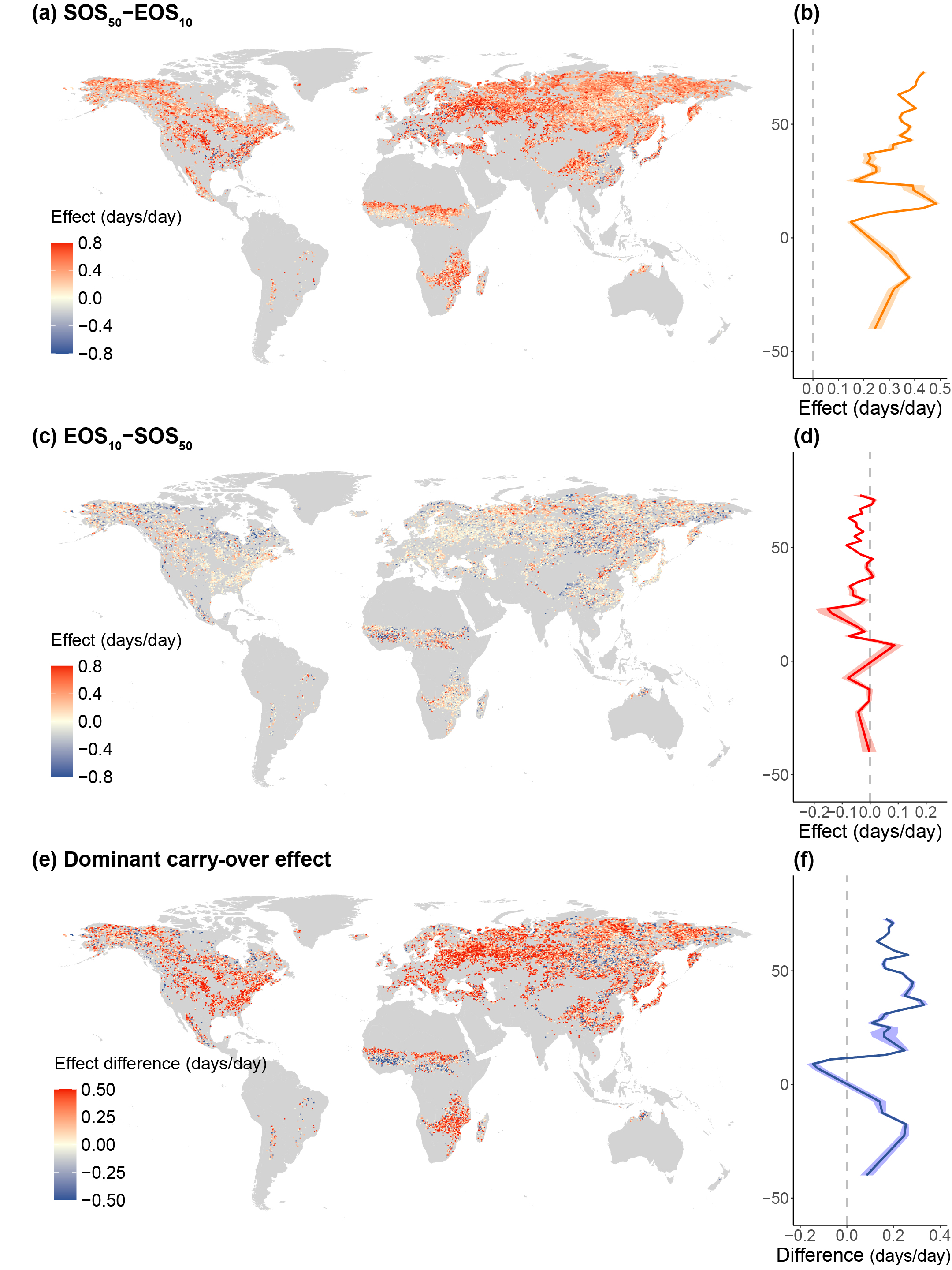


**Fig. S7. Global carry-over effects between mid leaf-out (SOS_50_) and leaf senescence onset (EOS_10_). a**, Map showing the effect of SOS_50_ on EOS_10_ [SOS-EOS effect] at a 0.25° resolution, derived from multilinear regression models with year and preseason temperature as covariates. **b**, Latitudinal variations in the SOS-EOS effect, with solid lines representing mean regression coefficients and shaded areas indicating standard deviations, summarized for each 2° latitude band (bands with fewer than 100 pixels were removed). **c**, **d**, Map and latitudinal variation for the effect of EOS_10_ on subsequent SOS_50_ (EOS-SOS effect). **e**, Map illustrating the dominant carry-over effect, calculated as the difference between the absolute coefficients of the SOS-EOS and EOS-SOS effects. Red pixels indicate regions where SOS-EOS effects dominate, while blue pixels indicate stronger EOS-SOS effects. **f**, Latitudinal variations in the relative importance of SOS-EOS versus EOS-SOS effects, with positive values reflecting stronger SOS-EOS effects and negative values indicating stronger EOS-SOS effects.


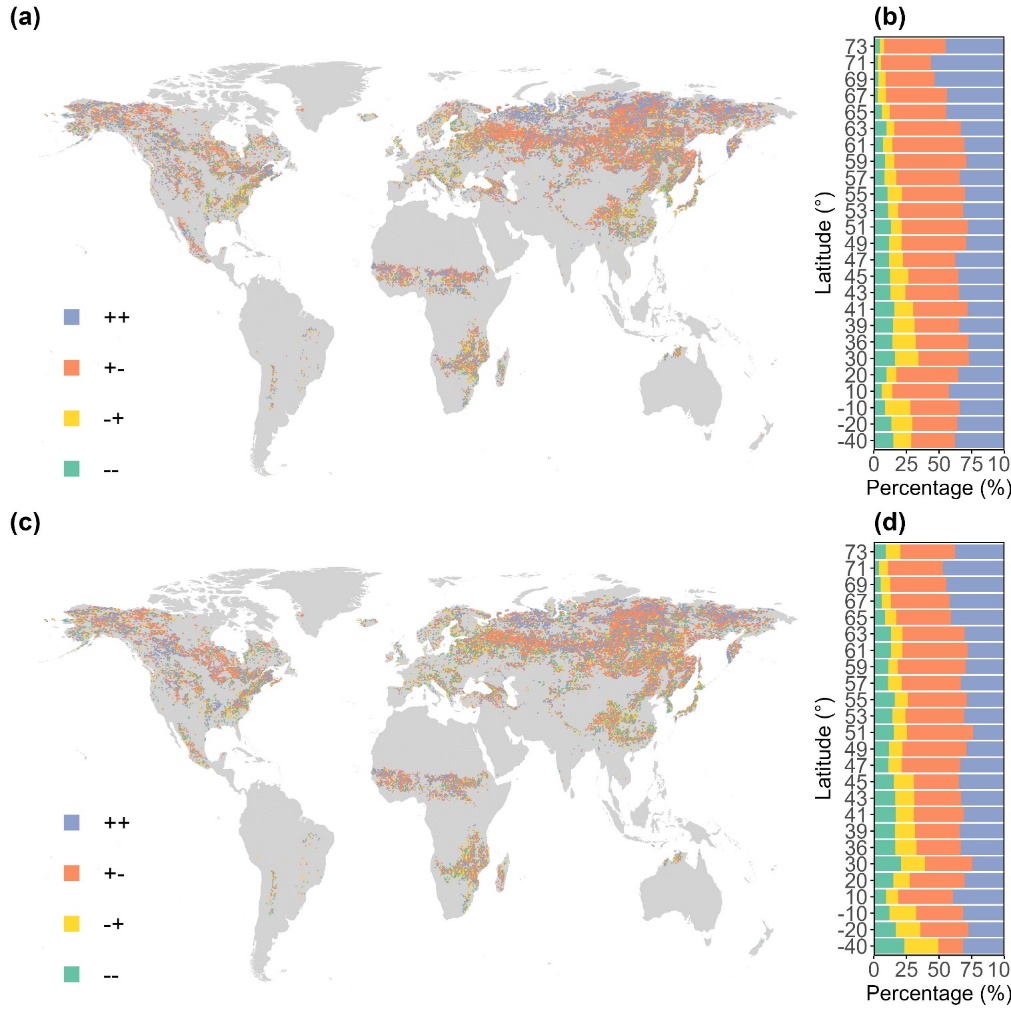


**Fig. S8. Distribution of phenological carry-over types. a,** Carry-over effects between leaf-out onset (SOS_15_) and leaf senescence onset (EOS_10_), classified based on the direction and significance (at *p* < 0.05) of each effect. Color codes indicate: "++" (both effects positive), "+-" (positive SOS-EOS effect and negative EOS-SOS effect), "-+" (negative SOS-EOS effect and positive EOS-SOS effect), and "--" (both effects negative). **b,** Latitudinal variations in the percentage of phenological carry-over types, summarized based on the pixels within each latitude band (bands with fewer than 100 pixels were removed).


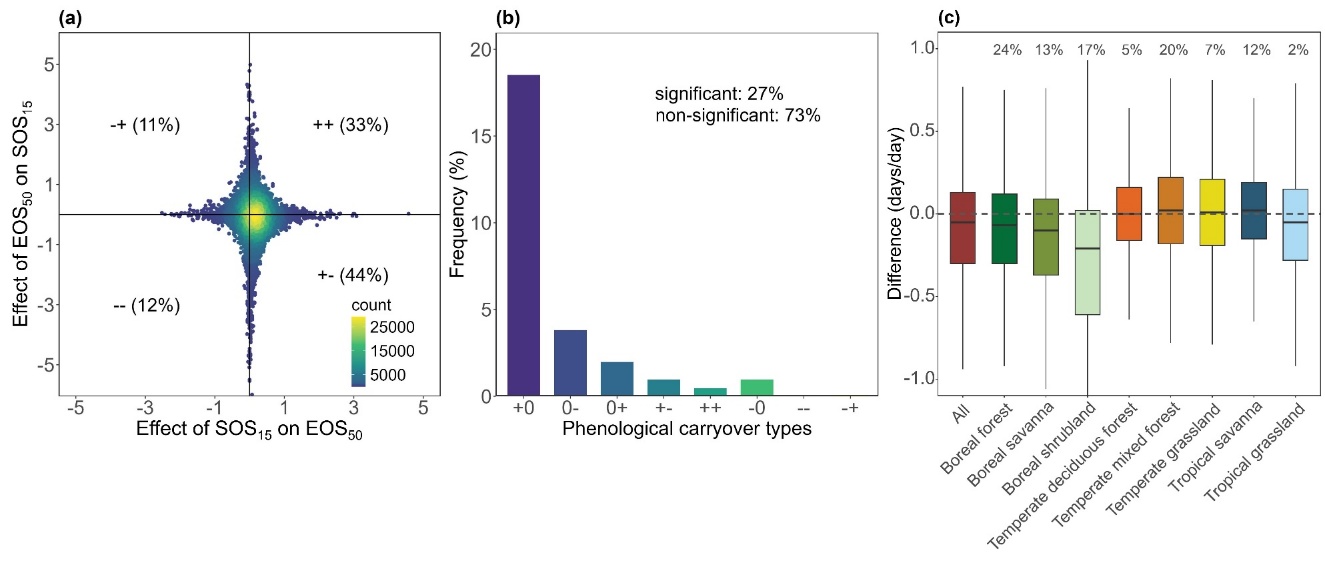


**Fig. S9. Relationship between the two phenological carry-over effects in seasonally deciduous vegetation (same as Fig. 3 but using EOS_50_ instead of EOS_10_ to represent autumn phenology). a,** The relationship between the SOS-EOS and EOS-SOS carry-over effects across all pixels. The SOS-EOS effect (SOS_15_ on EOS_50_) and EOS-SOS effect (EOS_50_ on SOS_15_) were estimated using multilinear regression models with year and preseason temperature as covariates. Percentages in each quadrant represent the proportion of pixels within that category. **b**, Frequency distribution of phenological carry-over types. The types are classified by the direction and significance (at *p* < 0.05) of each carry-over effect. For instance, "++" indicates both effects are positive, while "+0" signifies a positive SOS-EOS effect and no significant EOS-SOS effect. Significant carry-over effects were observed in 27% of pixels.


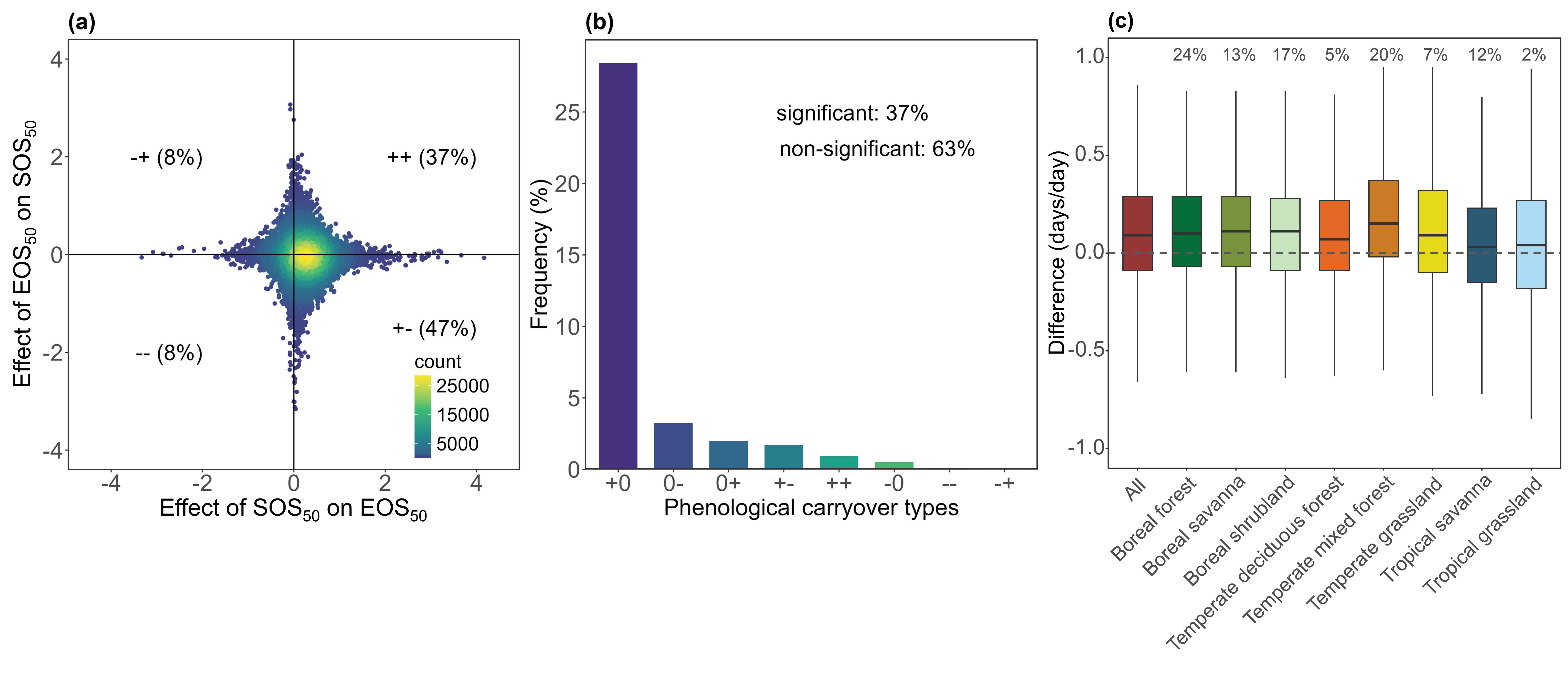


**Fig. S10. Relationship between the two phenological carry-over effects in seasonally deciduous vegetation (same as Fig. S3 but using SOS_50_ instead of SOS_15_ to represent spring phenology and using EOS_50_ instead of EOS_10_ to represent autumn phenology). a,** The relationship between the SOS-EOS and EOS-SOS carry-over effects across all pixels. The SOS-EOS effect (SOS_50_ on EOS_50_) and EOS-SOS effect (EOS_50_ on SOS_50_) were estimated using multilinear regression models, with year and preseason temperature as covariates. Percentages in each quadrant represent the proportion of pixels within that category. **b**, Frequency distribution of phenological carry-over types. The types are classified by the direction and significance (at *p* < 0.05) of each carry-over effect. For instance, "++" indicates that both effects are positive, while "+0" signifies a positive SOS-EOS effect and a non-significant EOS-SOS effect. Significant carry-over effects were observed in 37% of pixels.


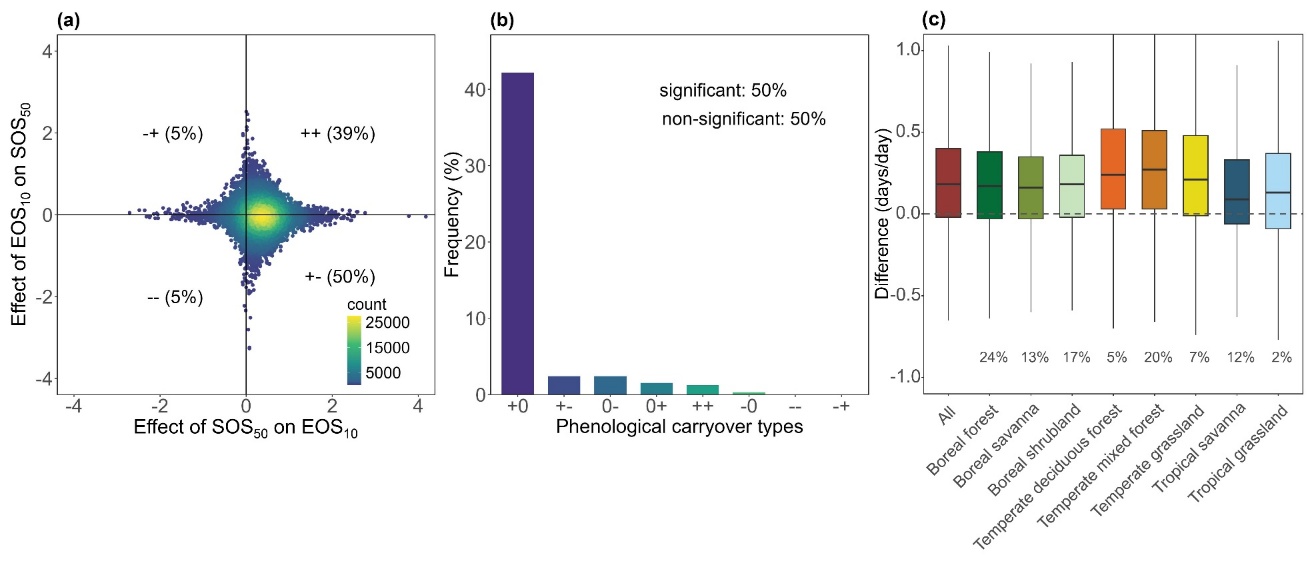


**Fig. S11. Relationship between the two phenological carry-over effects in seasonally deciduous vegetation (same as Fig. 3 but using SOS_50_ instead of SOS_10_ to represent spring phenology). a,** The relationship between the SOS-EOS and EOS-SOS carry-over effects across all pixels. The SOS-EOS effect (SOS_50_ on EOS_10_) and EOS-SOS effect (EOS_10_ on SOS_50_) were estimated using multilinear regression models with year and preseason temperature as covariates. Percentages in each quadrant represent the proportion of pixels within that category. **b**, Frequency distribution of phenological carry-over types. The types are classified by the direction and significance (at *p* < 0.05) of each carry-over effect. For instance, "++" indicates both effects are positive, while "+0" signifies a positive SOS-EOS effect and no significant EOS-SOS effect. Significant carry-over effects were observed in 50% of pixels.

**
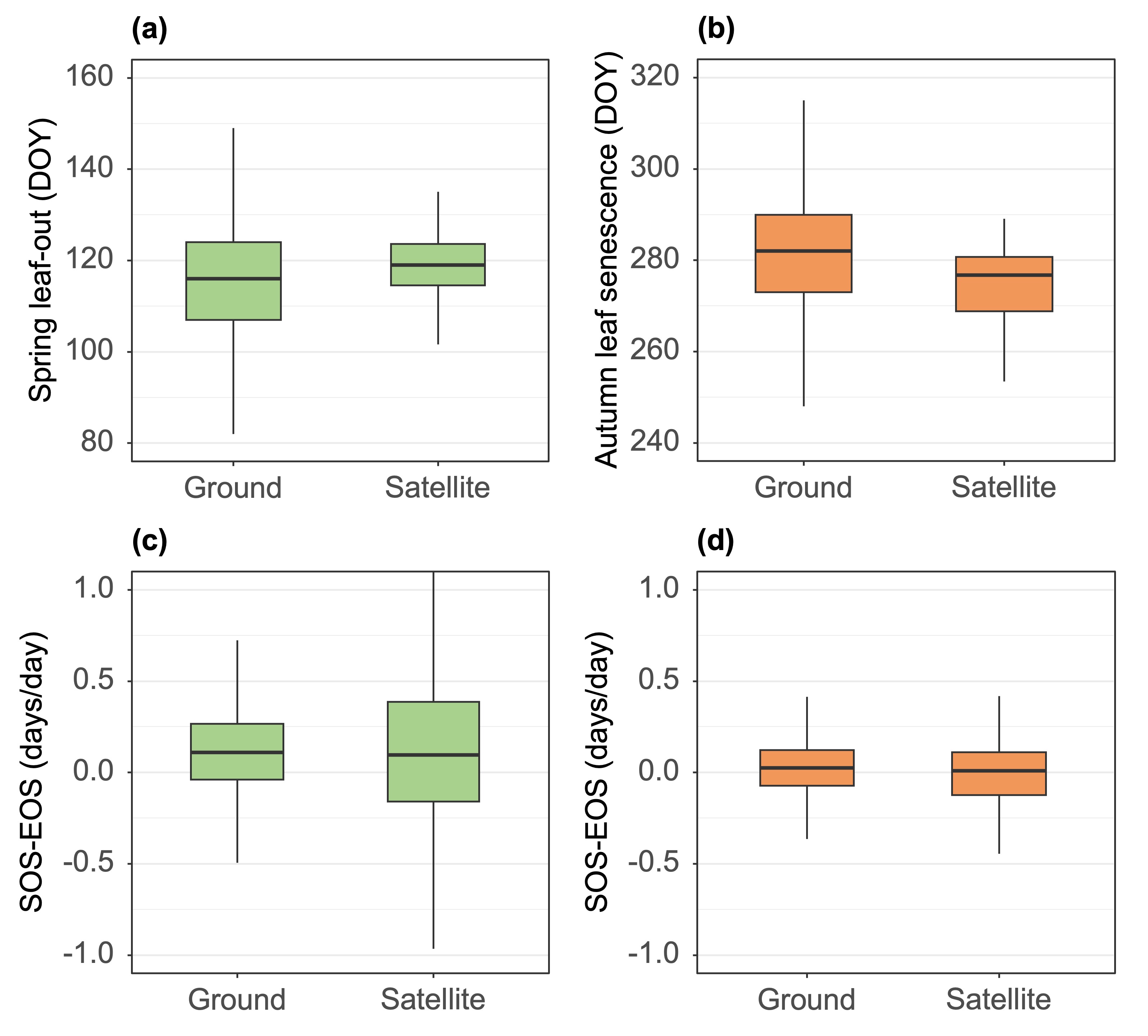
**

**Fig. S12. Comparison of leaf-out and leaf senescence timing (a, b) and phenological carry-over effects (c, d) between ground-based and satellite observations.** Satellite-derived forest phenology metrics were extracted from the main regions covered by the PEP725 network, while ground-based observations represent averages across all sites and species. SOS-EOS represents the effect of mid leaf-out (SOS_50_) on mid-senescence (EOS_50_), whereas EOS-SOS indicates the effect of EOS_50_ on the subsequent SOS_50_.


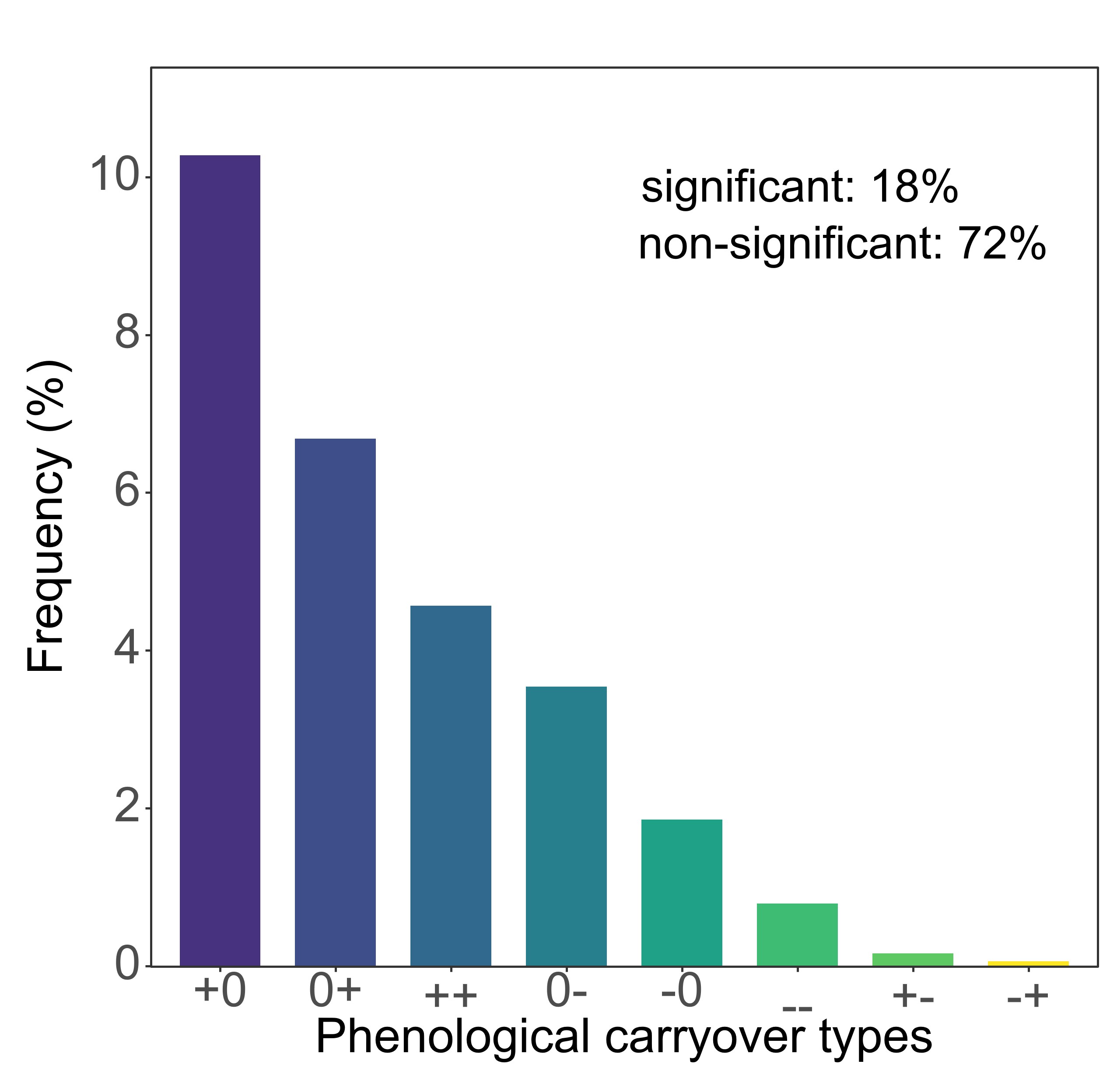


**Fig. S13. Relationship between the two phenological carry-over effects based on ground-based dataset.** The SOS-EOS effect (leaf-out on senescence) and EOS-SOS effect (leaf senescence on leaf-out) were estimated using multilinear regression models, with year and preseason temperature as covariates. The phenological carry-over types are classified by the direction and significance (at *p* < 0.05) of each carry-over effect. For instance, "++" indicates both effects are positive, while "+0" signifies a positive SOS-EOS effect and no significant EOS-SOS effect. Significant carry-over effects were observed in 18% of site-species combinations.


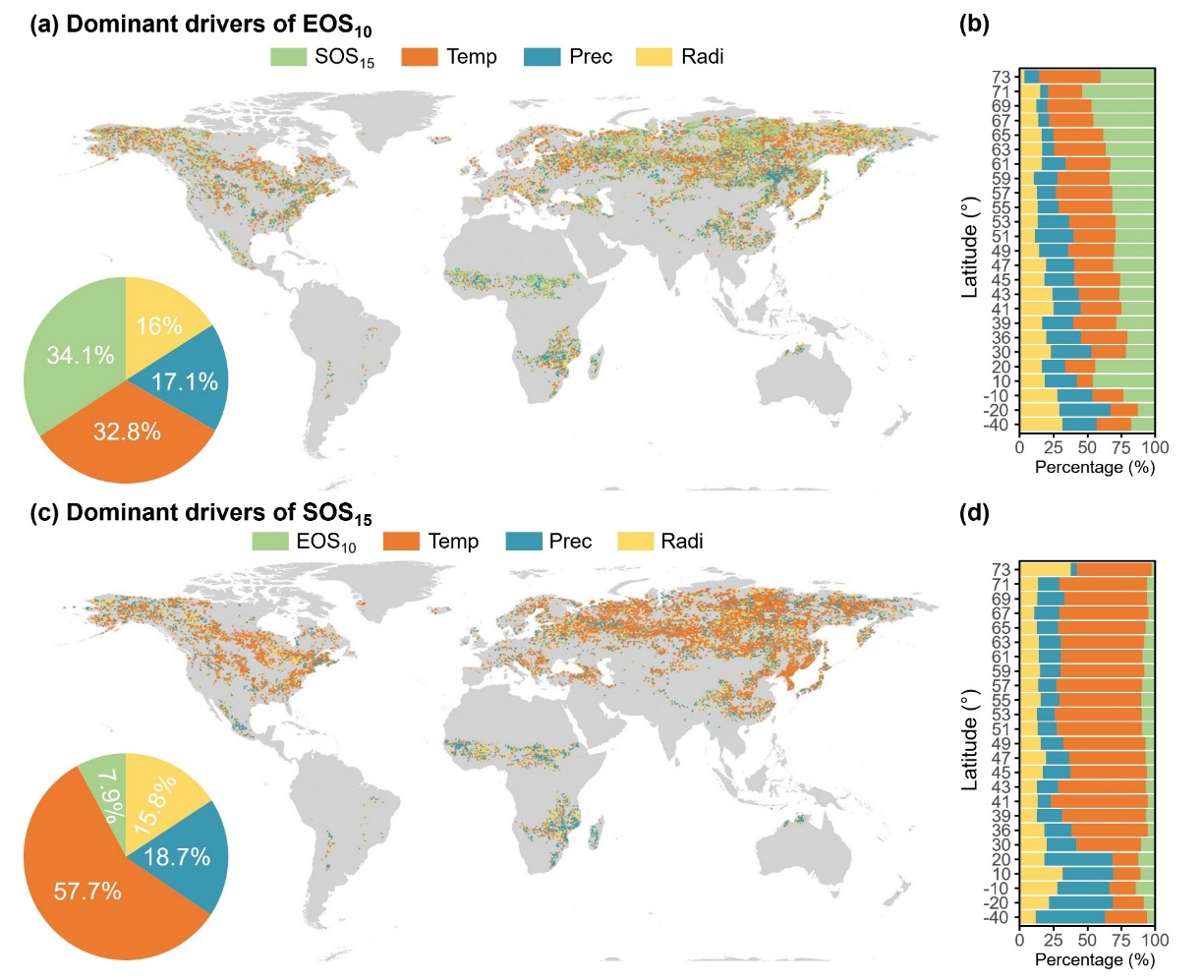


**Fig. S14. Predictors with the strongest statistical significance for leaf senescence onset (EOS_10_) and leaf-out onset (SOS_15_) across seasonal vegetation. a**, The best predictor of EOS_10_ for each pixel, determined from multilinear regression models that include SOS_15_, preseason temperature (Temp), precipitation (Prec), and radiation (Radi) as covariates. The best predictor is identified as the factor with the smallest *p*-value for its regression coefficient. The pie chart shows the proportion of pixels where each factor dominates. **b,** Latitudinal variation in the percentage of the best predictor of EOS_10_, summarized based on the pixels within each latitude band (bands with fewer than 100 pixels were removed). **c**, The best predictor of SOS_15_ for each pixel, based on models incorporating EOS_10_, preseason temperature, precipitation, and radiation. The dominant predictor is similarly determined by the smallest *p*-value. The pie chart illustrates the proportion of pixels where each factor is dominant. **d,** Latitudinal variation in the percentage of the best predictor of SOS_15_, summarized based on the pixels within each latitude band (bands with fewer than 100 pixels were removed).


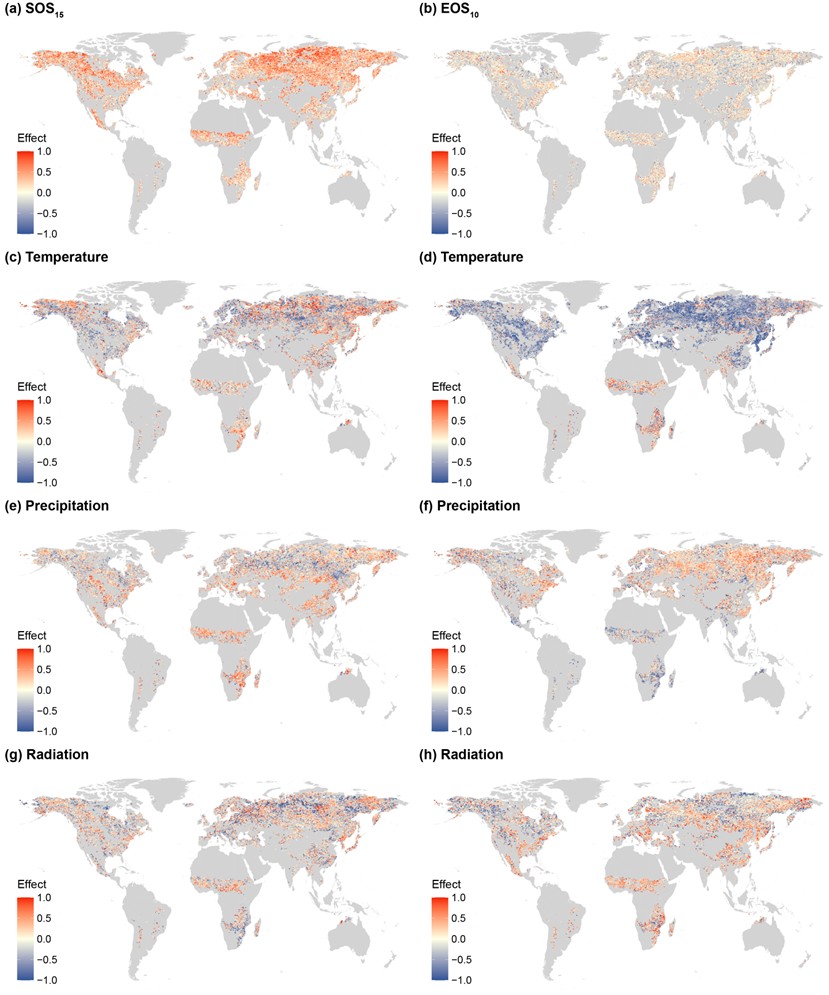


**Fig. S15. Carry-over effects and environmental factors in driving leaf senescence onset (EOS_10_) and leaf-out onset (SOS_15_). a**, **c**, **e**, **g**, Standardized effects of SOS_15_, temperature, precipitation and radiation on EOS_10_ for all pixels. **b**, **d**, **f**, **h**, Standardized effects of EOS_10_, temperature, precipitation and radiation on SOS_15_ for all pixels.


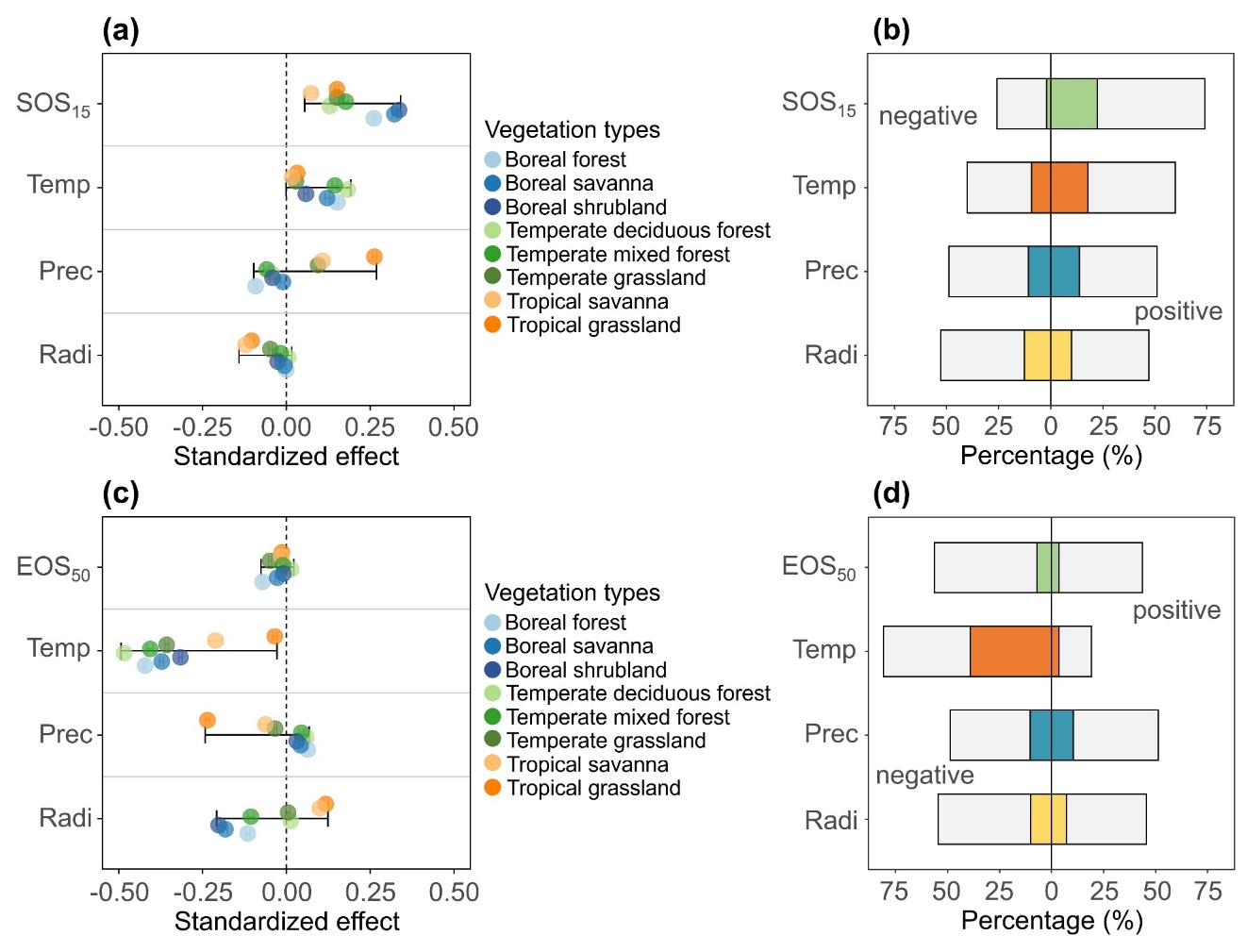


**Fig. S16. Relative contributions of carry-over effect and environmental factors in driving mid-senescence (EOS_50_) and leaf-out onset (SOS_15_). a, c,** Standardized effect sizes of predictor variables—SOS_15_ (**a**) or EOS_50_ (**c**) (carry-over effect), preseason temperature (Temp), precipitation (Prec), and radiation (Radi)—on EOS_50_ (**a**) and SOS_15_ (**c**), estimated from Bayesian linear mixed-effects models fitted separately for each vegetation type. All predictors were standardized prior to modeling. Pixel was specified as a random effect, and year was modeled as a random slope within pixels to account for temporal variability. Colored points represent posterior means for each vegetation type; black error bars denote the posterior mean and 95% credible interval of biome-level average effects. **b, d**, Percentage of pixels where each predictor influenced EOS_50_ (**b**) or SOS_15_ (**d**), based on multiple linear regression models. Positive and negative bars represent the percentage of pixels with positive and negative effects, respectively. Colored bars indicate the percentage of significant pixels (*p* < 0.05) for each variable; gray bars show the proportion of non-significant pixels.


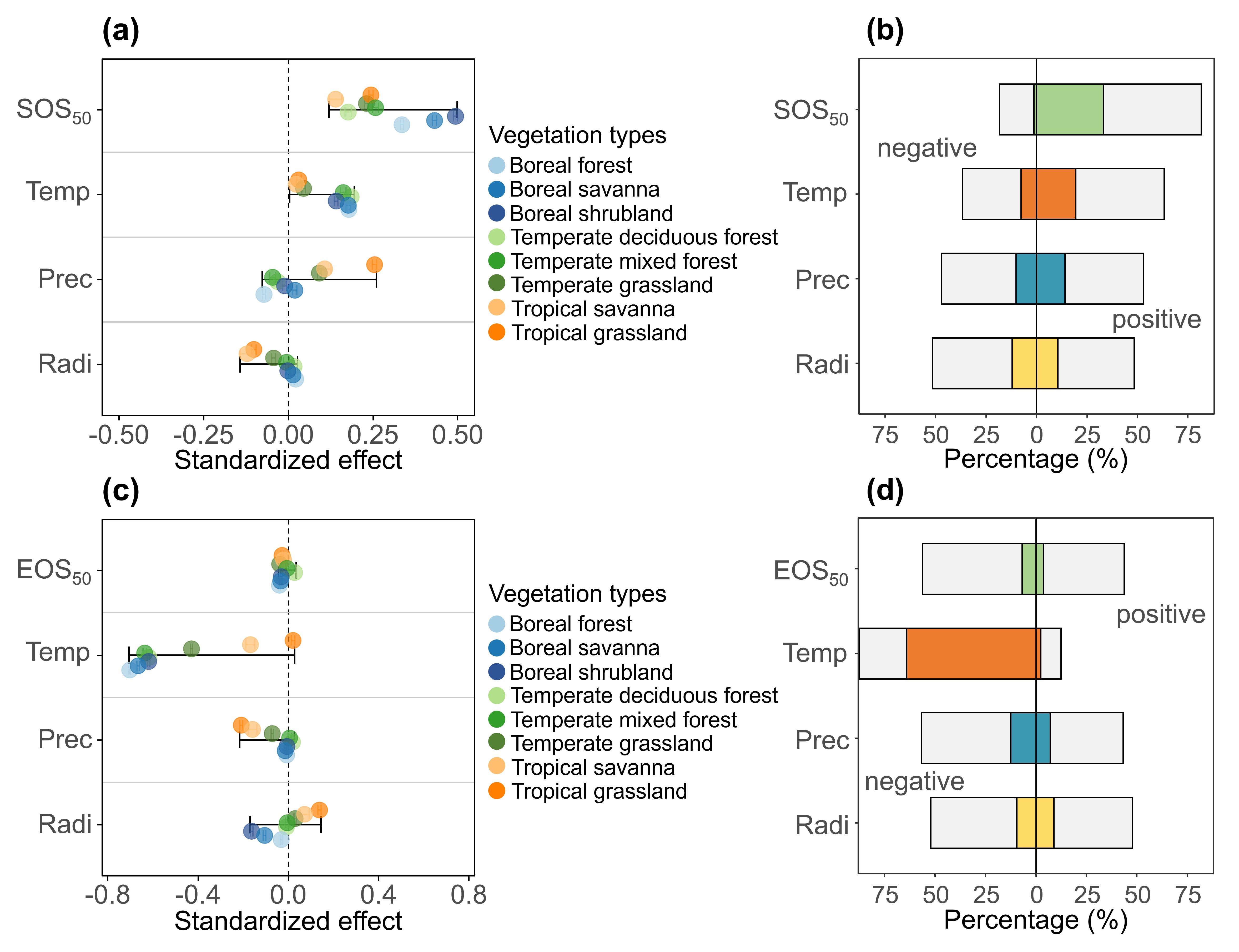


**Fig. S17. Relative contributions of carry-over effects and environmental factors in driving mid-senescence (EOS_50_) and mid leaf-out (SOS_50_).** **a, c,** Standardized effect sizes of predictor variables—SOS_50_ (**a**) or EOS_50_ (**c**) (carry-over effect), preseason temperature (Temp), precipitation (Prec), and radiation (Radi)—on EOS_50_ (**a**) and SOS_50_ (**c**), estimated from Bayesian linear mixed-effects models fitted separately for each vegetation type. All predictors were standardized prior to modeling. Pixel was specified as a random effect, and year was modeled as a random slope within pixels to account for temporal variability. Colored points represent posterior means for each vegetation type; black error bars denote the posterior mean and 95% credible interval of biome-level average effects. **b, d**, Percentage of pixels where each predictor influenced EOS_50_ (**b**) or SOS_50_ (**d**), based on multiple linear regression models. Positive and negative bars represent the percentage of pixels with positive and negative effects, respectively. Colored bars indicate the percentage of significant pixels (*p* < 0.05) for each variable; gray bars show the proportion of non-significant pixels.


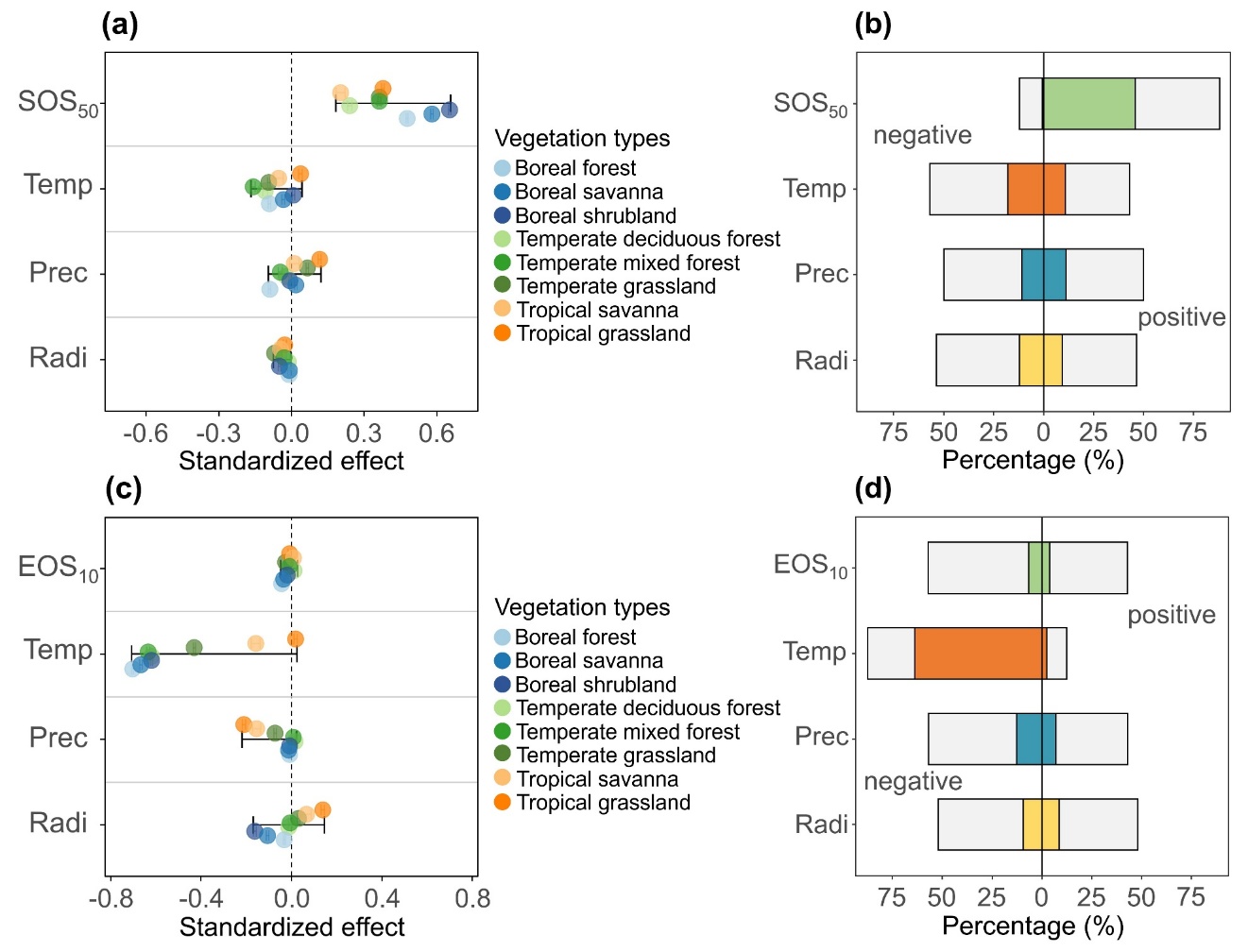


**Fig. S18. Relative contributions of carry-over effects and environmental factors in driving senescence onset (EOS_10_) and mid leaf-out (SOS_50_). a, c,** Standardized effect sizes of predictor variables—SOS_50_ (**a**) or EOS_10_ (**c**) (carry-over effect), preseason temperature (Temp), precipitation (Prec), and radiation (Radi)—on EOS_10_ (**a**) and SOS_50_ (**c**), estimated from Bayesian linear mixed-effects models fitted separately for each vegetation type. All predictors were standardized prior to modeling. Pixel was specified as a random effect, and year was modeled as a random slope within pixels to account for temporal variability. Colored points represent posterior means for each vegetation type; black error bars denote the posterior mean and 95% credible interval of biome-level average effects. **b, d**, Percentage of pixels where each predictor influenced EOS_10_ (**b**) or SOS_50_ (**d**), based on multiple linear regression models. Positive and negative bars represent the percentage of pixels with positive and negative effects, respectively. Colored bars indicate the percentage of significant pixels (*p* < 0.05) for each variable; gray bars show the proportion of non-significant pixels.


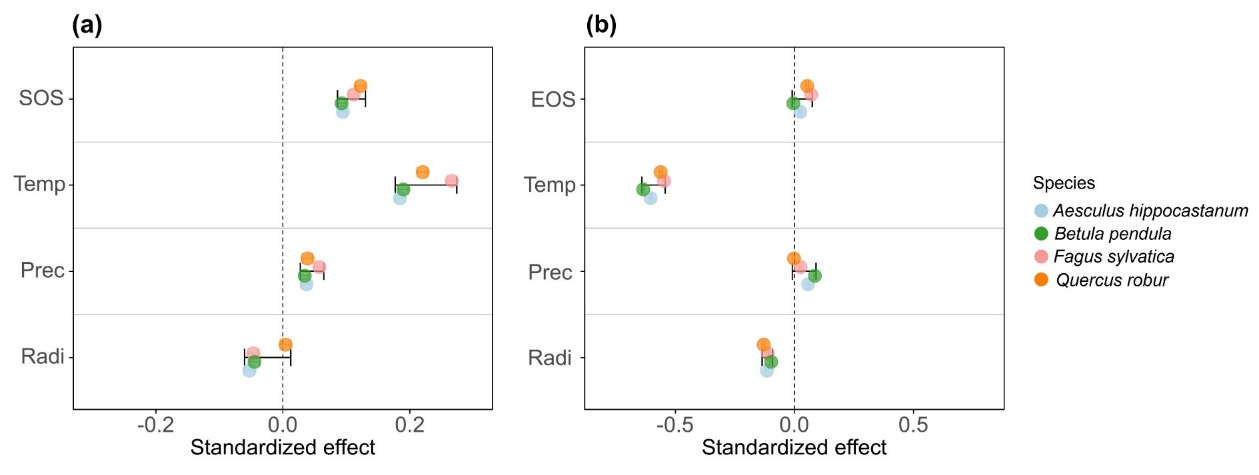


**Fig. S19. Relative contributions of carry-over effects and environmental factors in driving leaf senescence (EOS) and leaf-out (SOS) based on ground-based dataset.** Standardized effect sizes of predictor variables—SOS (**a**) or EOS (**c**) (carry-over effects), preseason temperature (Temp), precipitation (Prec), and radiation (Radi)—on EOS (**a**) and SOS (**b**), estimated from Bayesian linear mixed-effects models fitted separately for each tree species. All predictors were standardized prior to modeling. Site was specified as a random effect, and year was modeled as a random slope within sites to account for temporal variability. Colored points represent posterior means for each tree species; black error bars denote the posterior mean and 95% credible interval of biome-level average effects.


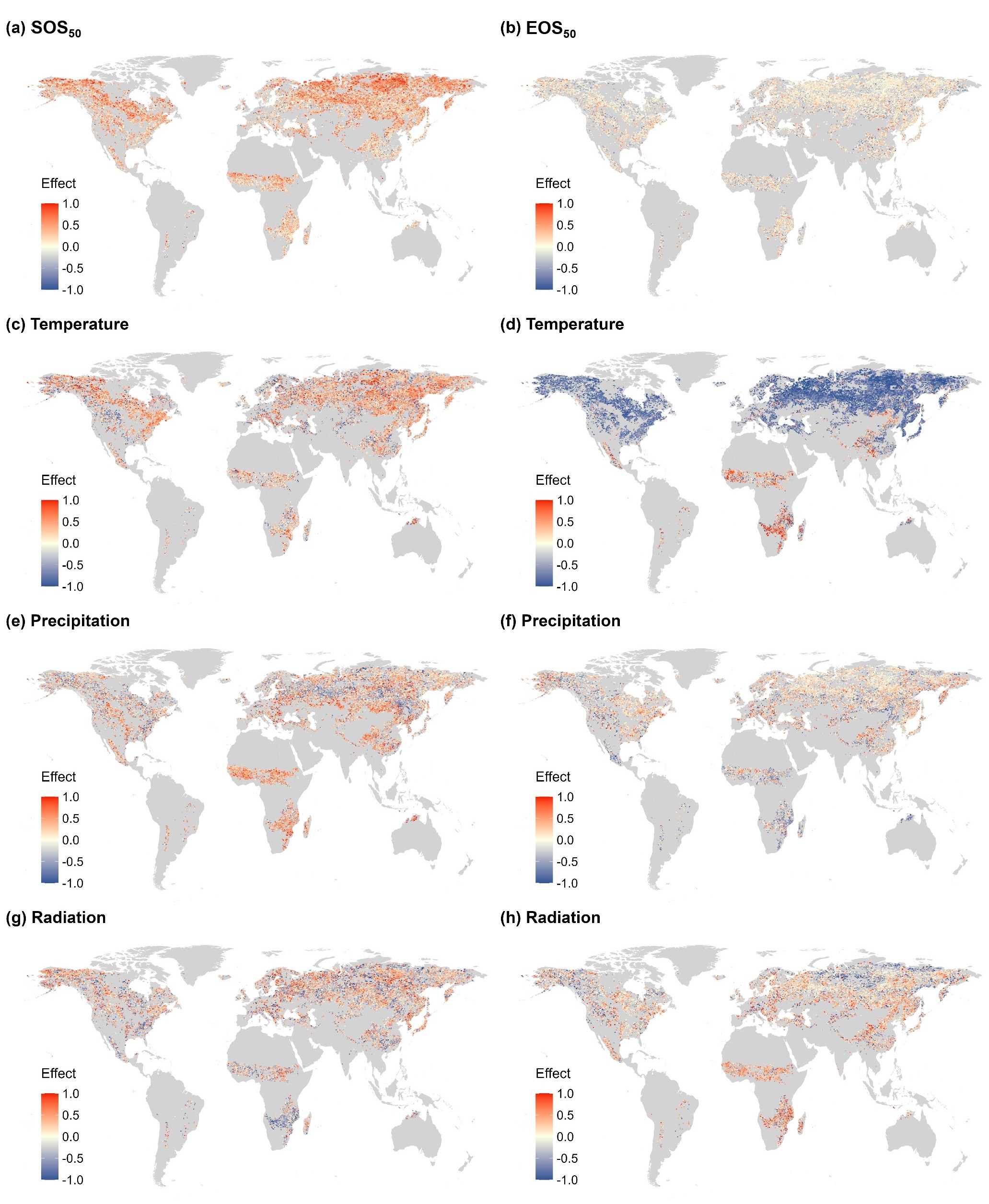


**Fig. S20. Carry-over effects and environmental factors in driving mid-senescence (EOS_50_) and mid leaf-out (SOS_50_). a**, **c**, **e**, **g**, Standardized effects of SOS_50_, temperature, precipitation and radiation on EOS_50_ for all pixels. **b**, **d**, **f**, **h**, Standardized effects of EOS_50_, temperature, precipitation and radiation on SOS_50_ for all pixels.


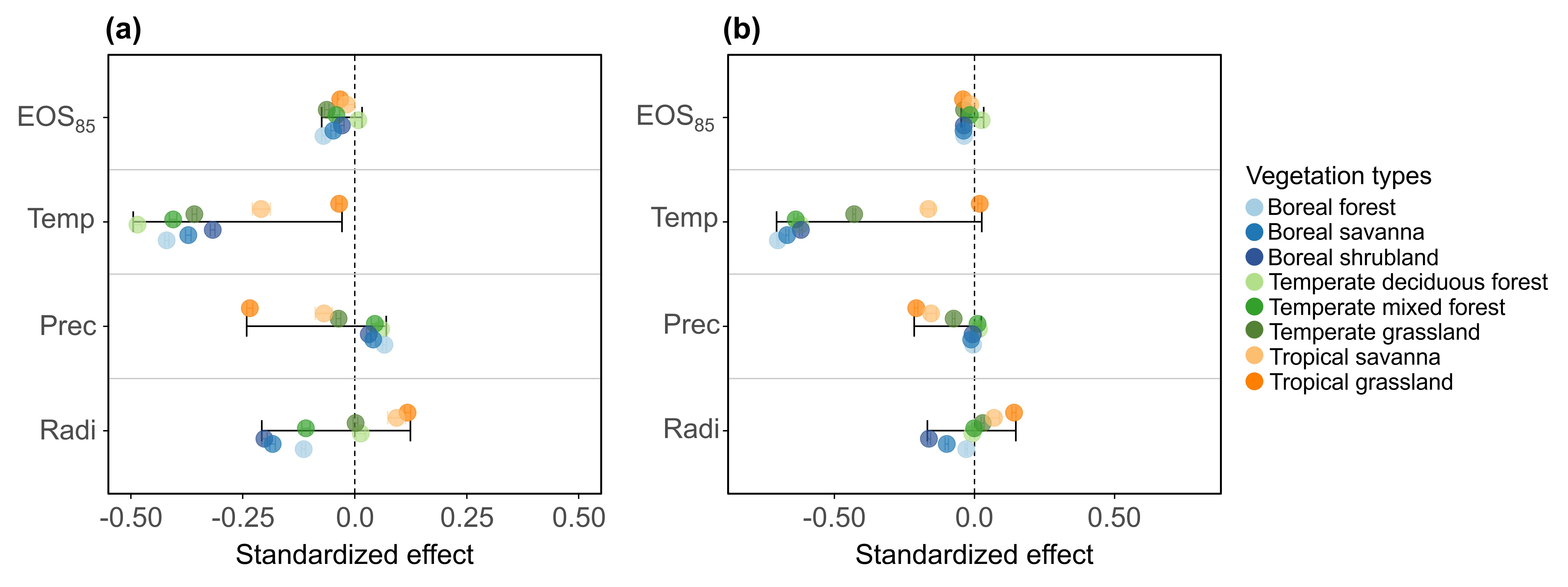


**Fig. S21. Relative contributions of late leaf senescence (EOS_85_) and environmental factors in driving the onset (SOS_15_, a) and mid (SOS_50_, b) of leaf-out.** Standardized effect sizes of predictor variables—EOS_85_ (carry-over effects), preseason temperature (Temp), precipitation (Prec), and radiation (Radi)—on SOS_15_ (**a**) and SOS_50_ (**b**), estimated from Bayesian linear mixed-effects models fitted separately for each vegetation type. All predictors were standardized prior to modeling. Pixel was specified as a random effect, and year was modeled as a random slope within pixels to account for temporal variability. Colored points represent posterior means for each vegetation type; black error bars denote the posterior mean and 95% credible interval of biome-level average effects.


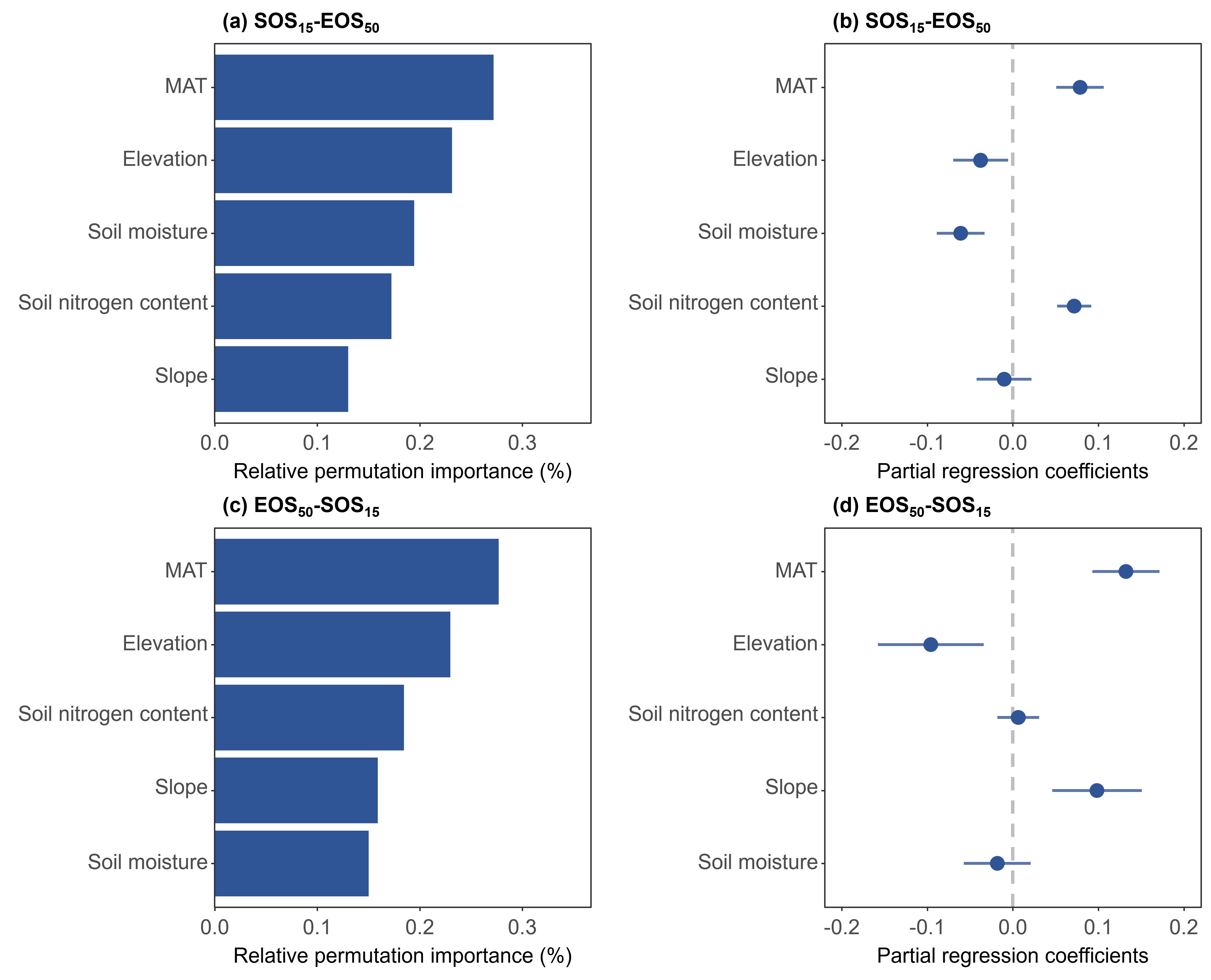


**Fig. S22. Environmental predictors of spatial variation in phenological carry-over effects on mid-senescence (EOS_50_) and leaf-out onset (SOS_15_). a**, **c**, Relative permutation importance of five environmental predictors in random forest models, ranked by their contribution to explaining spatial variation in the SOS-EOS effect (**a**) and EOS-SOS effect (**c**). The SOS-EOS effect quantifies the influence of SOS_15_ on EOS_50_, while the EOS-SOS effect captures the influence of EOS_50_ on the following year’s SOS_15_. **b**, **d**, Bootstrapped partial regression coefficients (mean ± sd) showing the linear influence of each predictor on the SOS-EOS (**b**) and EOS-SOS (**d**) effects. Coefficients were averaged across 100 multivariate linear models. All variables were standardized to allow direct comparison of effect sizes. Predictors include mean annual temperature (MAT), soil moisture, elevation, soil nitrogen content and slope.


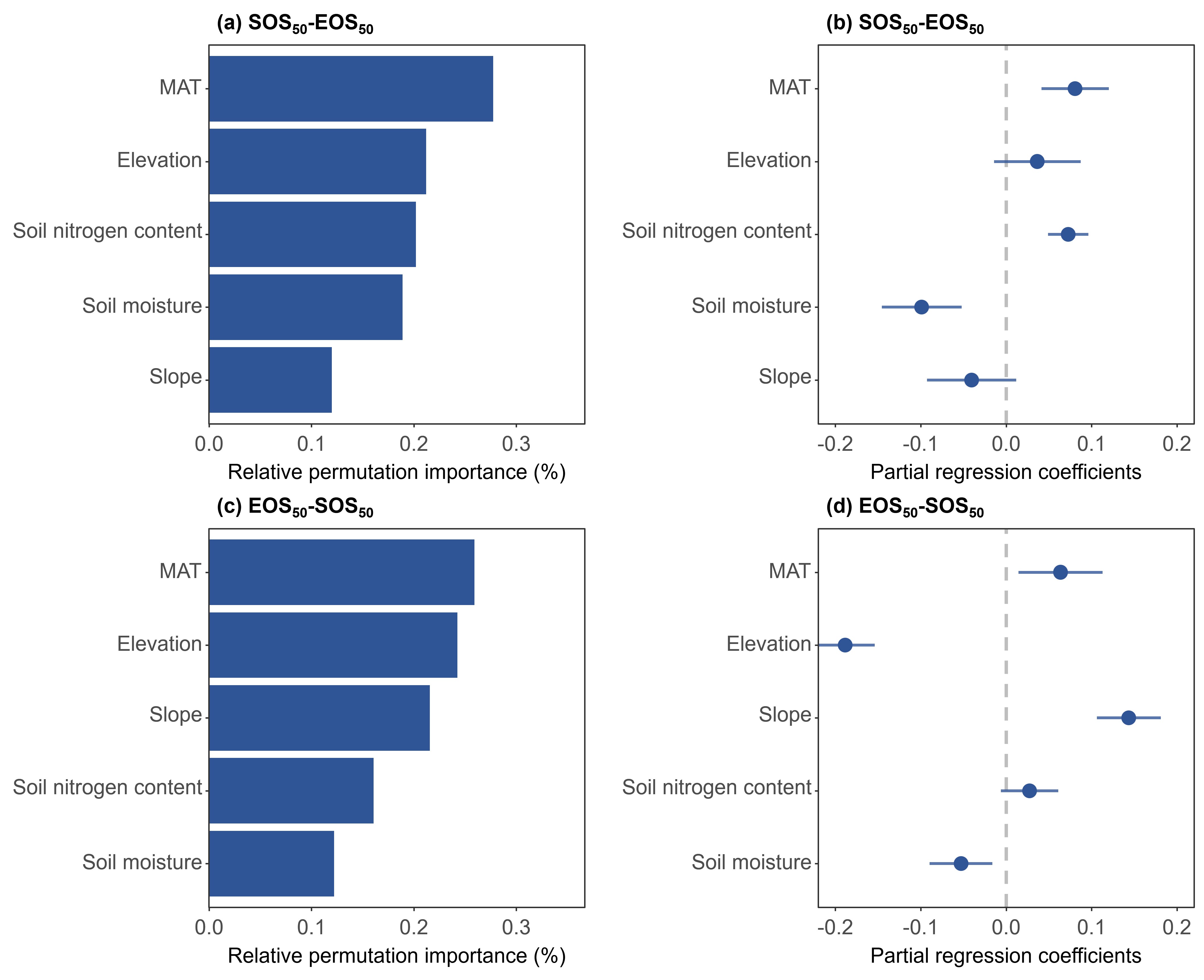


**Fig. S23. Environmental predictors of spatial variation in phenological carry-over effects on mid-senescence (EOS_50_) and mid leaf-out (SOS_50_). a**, **c**, Relative permutation importance of five environmental predictors in random forest models, ranked by their contribution to explaining spatial variation in the SOS-EOS effect (**a**) and EOS-SOS effect (**c**). The SOS-EOS effect quantifies the influence of SOS_50_ on EOS_50_, while the EOS-SOS effect captures the influence of EOS_50_ on the following year’s SOS_50_. **b**, **d**, Bootstrapped partial regression coefficients (mean ± sd) showing the linear influence of each predictor on the SOS-EOS (**b**) and EOS-SOS (**d**) effects. Coefficients were averaged across 100 multivariate linear models. All variables were standardized to allow direct comparison of effect sizes. Predictors include mean annual temperature (MAT), soil moisture, elevation, soil nitrogen content and slope.


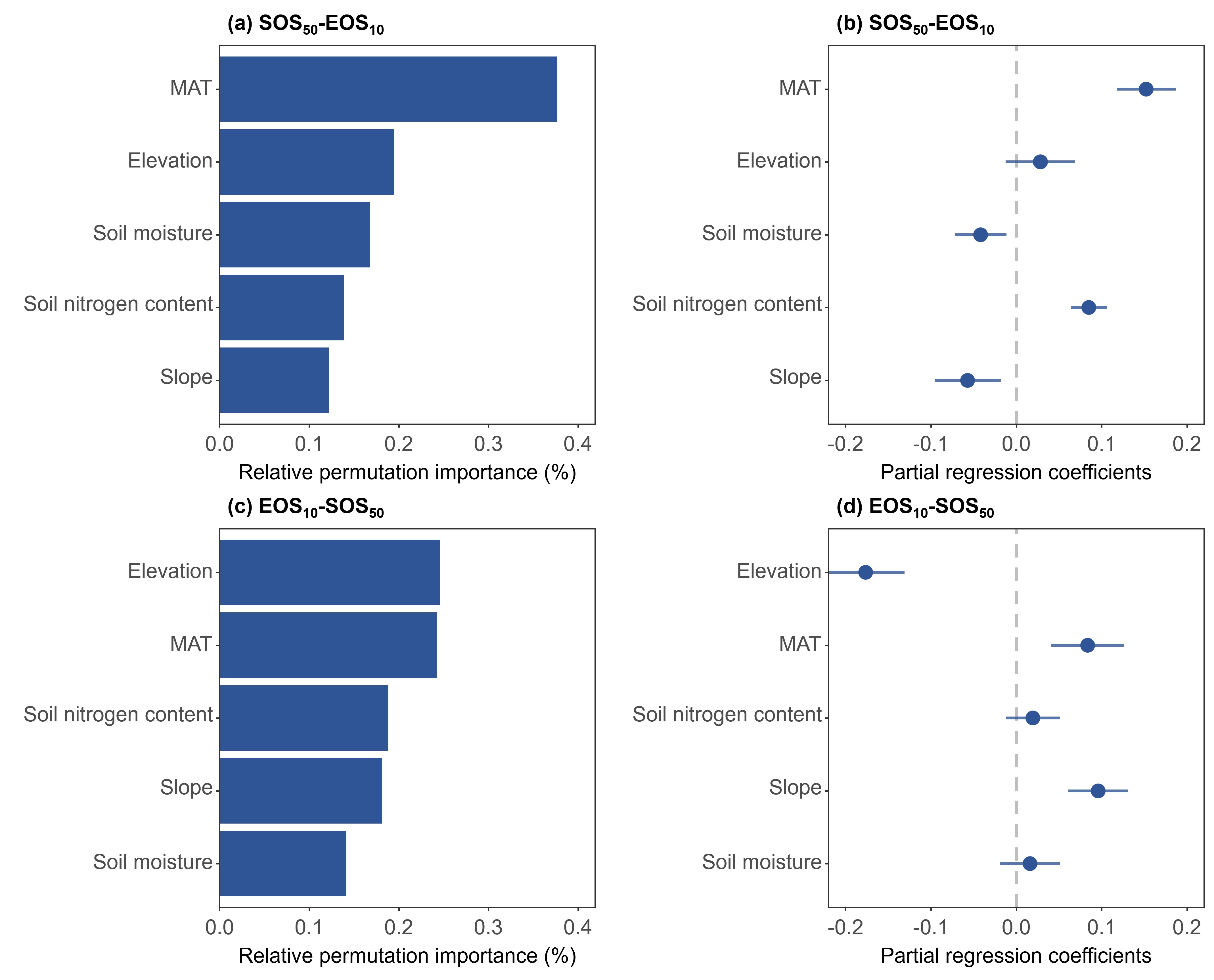


**Fig. S24. Environmental predictors of spatial variation in phenological carry-over effects on senescence onset (EOS_10_) and mid leaf-out (SOS_50_). a**, **c**, Relative permutation importance of five environmental predictors in random forest models, ranked by their contribution to explaining spatial variation in the SOS-EOS effect (**a**) and EOS-SOS effect (**c**). The SOS-EOS effect quantifies the influence of SOS_50_ on EOS_10_, while the EOS-SOS effect captures the influence of EOS_10_ on the following year’s SOS_50_. **b**, **d**, Bootstrapped partial regression coefficients (mean ± sd) showing the linear influence of each predictor on the SOS-EOS (**b**) and EOS-SOS (**d**) effects. Coefficients were averaged across 100 multivariate linear models. All variables were standardized to allow direct comparison of effect sizes. Predictors include mean annual temperature (MAT), soil moisture, elevation, soil nitrogen content and slope.
